# Spiral Random Walk-Enhanced Puma Optimizer for Side Lobe Suppression and Null Control in Array Antenna Design

**DOI:** 10.3390/biomimetics11080584

**Published:** 2026-08-17

**Authors:** Ridvan Firat Cinar

**Affiliations:** Department of Computer Engineering, Faculty of Engineering and Architecture, Batman University, 72100 Batman, Turkey; ridvanfirat.cinar@batman.edu.tr

**Keywords:** linear antenna array, pattern nulling, null steering optimization, metaheuristics, puma optimizer

## Abstract

This study proposes a spiral random walk enhanced puma optimizer (SRW-PO) for array antenna design. The method preserves the hunting and ambush search structure of the classical puma optimizer (PO) while embedding two complementary mechanisms into its update process. The adaptive random walk operator improves population diversity and enables weak candidate solutions to escape unproductive regions. The spiral local search operator strengthens exploitation around the current best solution. This structure provides a balanced search process for highly nonlinear antenna synthesis problems involving side lobe suppression, null control, and excitation dynamic range. SRW-PO is evaluated on representative linear array antenna design cases and further assessed through a focused CEC2022 benchmark comparison with classical PO, GWO, WOA, and GA. The results indicate stronger convergence, more consistent objective cost reduction, and competitive computational efficiency. In the CEC2022 benchmark, SRW-PO achieves the best average rank and ranks first for most benchmark functions. In antenna synthesis experiments, it provides accurate radiation pattern shaping, effective side lobe reduction, and deep null placement. These findings indicate that the embedded spiral and random walk mechanisms improve the search quality and optimization consistency of classical PO for array antenna design.

## 1. Introduction

### 1.1. State of the Art in Antenna Array Synthesis

Antenna array design addresses the engineering problem of shaping radiation patterns in the presence of interference, coupled angular requirements, and hardware constraints. In wireless communication, radar, satellite systems, remote sensing, and surveillance, arrays act as spatial filters that enhance desired directions and attenuate unwanted signals [1]. Their radiation characteristics are governed by excitation amplitudes, excitation phases, element locations, and inter-element spacing [2]. Practical synthesis therefore requires simultaneous side lobe suppression, prescribed null placement, deep interference attenuation, main beam preservation, half-power beamwidth control, and feasible excitation amplitudes [3]. These objectives are coupled rather than independent. For example, increasing null depth at an interference direction can raise side lobes, distort the main beam, broaden the beamwidth, or increase amplitude variation across the aperture. Interference-aware array synthesis is consequently a constrained multi-objective problem in which electromagnetic performance and implementation complexity must be considered together.

Classical analytical techniques provide structured solutions for standard array pattern requirements. Dolph–Chebyshev synthesis derives broadside distributions that control the equal ripple side lobe level for a prescribed beamwidth trade-off [4]. Taylor synthesis provides line source distributions with a controlled near-in side lobe region and a gradual side lobe decay [5]. Fourier-based synthesis relates the desired angular response to an aperture distribution through spectral methods [6]. The Woodward–Lawson procedure constructs an aperture from sampled pattern specifications [7]. Schelkunoff polynomial methods formulate linear array synthesis through the placement of array factor roots [8]. These methods remain useful because their parameters have clear physical interpretations and their side lobe behavior is predictable. Their limitation appears when arbitrary deep nulls, multiple nulls, broad-null sectors, side lobe masks, and excitation dynamic range constraints must be satisfied simultaneously. Under such conditions, the required pattern may no longer correspond to a convenient analytical distribution.

Deterministic numerical methods extend the ability to impose explicit pattern constraints. Least squares synthesis minimizes the deviation between a desired response and the realized array pattern over selected angles [9]. Constrained nonlinear and gradient-based optimization methods permit direct treatment of bound constraints and differentiable penalty functions [10,11]. Convex optimization, quadratic programming, and second-order cone programming provide tractable formulations when the array variables and constraints can be expressed in compatible forms [12]. In antenna applications, convex formulations have been used to synthesize array patterns under specified side lobe and angular constraints [13]. However, deep nulling, dB-domain errors, amplitude-only control, broad-null sectors, and dynamic-range penalties can produce nonlinear, multimodal, non-smooth, or nonconvex objectives. Gradient-based methods may then depend on initialization and local curvature, while convex methods may require approximations or problem-specific reformulations that restrict the original design space [12].

### 1.2. Relevant Bio-Inspired Optimization Studies

Population-based metaheuristics offer derivative-free alternatives for such cases. Within evolutionary computation, the genetic algorithm represents adaptive search through selection and recombination mechanisms [14], whereas differential evolution uses vector-difference mutation to explore continuous spaces [15]. Within swarm intelligence, particle swarm optimization (PSO) updates candidate solutions through individual and collective search information [16], while artificial bee colony (ABC) models the distributed search behavior of honey bee colonies [17]. Animal- and nature-inspired approaches include grey wolf optimizer (GWO) [18], whale optimization algorithm (WOA) [19], Harris hawks optimization (HHO) [20], Schrödinger optimization [21], and moth–flame optimization (MFO) [22]. These methods have been applied to antenna synthesis for thinned arrays [23], phased-array pattern design [24], null-controlled linear-array geometry synthesis [25], and broader electromagnetic optimization tasks [26]. Their main advantage is that they can accommodate nonlinear objective functions and mixed constraints without requiring derivative information or a convex reformulation.

This flexibility does not remove the exploration–exploitation conflict. Excessive exploration may generate random movement with limited improvement, whereas early exploitation may reduce population diversity and cause premature convergence. Stagnation can occur when candidate solutions concentrate around a locally attractive pattern that does not satisfy all side lobe, null depth, beamwidth, and amplitude-range requirements. This issue is important in amplitude-only array synthesis because a small change in one coefficient affects the full angular response. An adjustment that improves a prescribed null may simultaneously increase the maximum side lobe level or worsen the excitation dynamic-range ratio. The resulting landscape contains coupled angular responses and several local minima, creating a need for search strategies that preserve diversity while refining the best regions found during optimization.

The puma optimizer (PO) is a population-based derivative-free method that models puma hunting behavior through global search and ambush-oriented exploitation [27]. Its search structure is suitable for bounded nonlinear problems in which analytical gradients are unavailable or unreliable. The present motivation is not that PO is generally inadequate. Rather, tightly constrained multimodal objectives can leave weak individuals in unproductive regions, reduce population diversity during later iterations, and provide insufficient inspection of the neighborhood surrounding the best solution. These conditions suggest two complementary modifications. The first should restore diversity and help weak candidates escape poor regions. The second should examine the vicinity of the current best candidate from several directions to improve local refinement.

Adaptive random walk provides a mechanism for reactivating weak candidates and promoting exploration beyond unproductive regions [28]. Its perturbation magnitude can decrease gradually as the search progresses, while increasing temporarily when stagnation indicates that the current population no longer generates useful alternatives [29]. Such a mechanism supports escape from unproductive regions without replacing the main PO search structure. Spiral movement provides a complementary local search behavior. By tracing candidate updates around the current best solution from changing directions, spiral motion inspects neighboring amplitude combinations more thoroughly than a purely direct movement [30]. Spiral search mainly strengthens exploitation, but it may be insufficient when population diversity has already collapsed [31]. The proposed spiral random walk-enhanced puma optimizer, SRW-PO, therefore, preserves the central PO backbone and embeds an adaptive random walk operator for weak candidates together with a spiral local search operator around the current best solution. The random walk operator supports diversity restoration and escape, whereas the spiral operator supports localized refinement.

The application considered in this study is the amplitude-only synthesis of a symmetric broadside linear array with 2N+1 isotropic elements and λ/2 spacing. Element positions and excitation phases remain fixed, while only real-valued symmetric amplitude coefficients are optimized. The objective function jointly treats null depth error, maximum side lobe-level error, and an excitation dynamic-range-ratio penalty. The dynamic-range-ratio is included because large amplitude variation increases the implementation burden of feed networks, attenuators, and amplitude-control hardware [2,32]. Classical methods have limited flexibility under these simultaneous constraints, and deterministic formulations may require restrictive assumptions. Metaheuristics offer a broader search capability but may lose diversity or provide weak local refinement. Within this antenna synthesis setting, the combined treatment of adaptive correction of weak individuals and spiral neighborhood refinement remains limited. Accordingly, this work develops SRW-PO by combining adaptive random walk and spiral local search while preserving the classical PO structure; formulates an amplitude-only linear-array model that jointly handles null depth, side lobe level, and excitation dynamic range; evaluates single-null, deep-null, multiple-null, and broad-null cases; and compares SRW-PO with PO, GA, WOA, and GWO under equal computational conditions and repeated independent runs. A focused CEC2022 benchmark is also used as complementary numerical evidence.

### 1.3. Research Gap and Main Contributions

Constrained amplitude-only antenna synthesis requires an optimization mechanism that can preserve population diversity, recover from stagnation, and refine promising solutions while jointly controlling null depth, side lobe level, and excitation dynamic range. This requirement motivates the integration of adaptive random walk and best centered spiral refinement into the PO framework.

The relevance of this study to biomimetics arises from its optimization methodology rather than from the antenna application itself. SRW-PO retains the animal-inspired foundation of the puma optimizer, which computationally abstracts the transition between wide-ranging search and ambush-oriented exploitation observed in puma hunting behavior. The embedded spiral refinement and adaptive random walk mechanisms strengthen neighborhood exploration and stagnation recovery without replacing this biomimetic search backbone. Antenna array synthesis is employed as a nonlinear and constrained engineering test case to demonstrate how an animal-inspired optimization principle can be transferred to electromagnetic design. Therefore, the contribution to biomimetics lies in the development and engineering evaluation of an enhanced biologically inspired search mechanism, while prescribed-null antenna synthesis provides its application and validation environment.

The main contributions are summarized as follows:

- Spiral refinement and adaptive random walk are embedded into the PO backbone to improve local search and diversity recovery.
- Null depth, side lobe level, and excitation DRR are jointly addressed under fixed phase amplitude-only synthesis.
- Single, deep, multiple, and broad-null scenarios are evaluated together with broadside gain-loss analysis.
- SRW-PO is assessed against PO, GA, WOA, and GWO using antenna applications, CEC2022 benchmarks, statistical analysis, computational complexity, and execution cost.

### 1.4. Paper Organization

The remainder of the paper is organized as follows. Section 2 presents the symmetric linear-array model and objective function. Section 3 describes classical PO and the SRW-PO operators. Section 4 reports the antenna-synthesis cases and comparative results. Section 5 presents the complementary CEC2022 evaluation, and Section 6 concludes the paper.

## 2. Mathematical Modelling of Amplitude-Controlled Array Nulling

**Figure 1:**
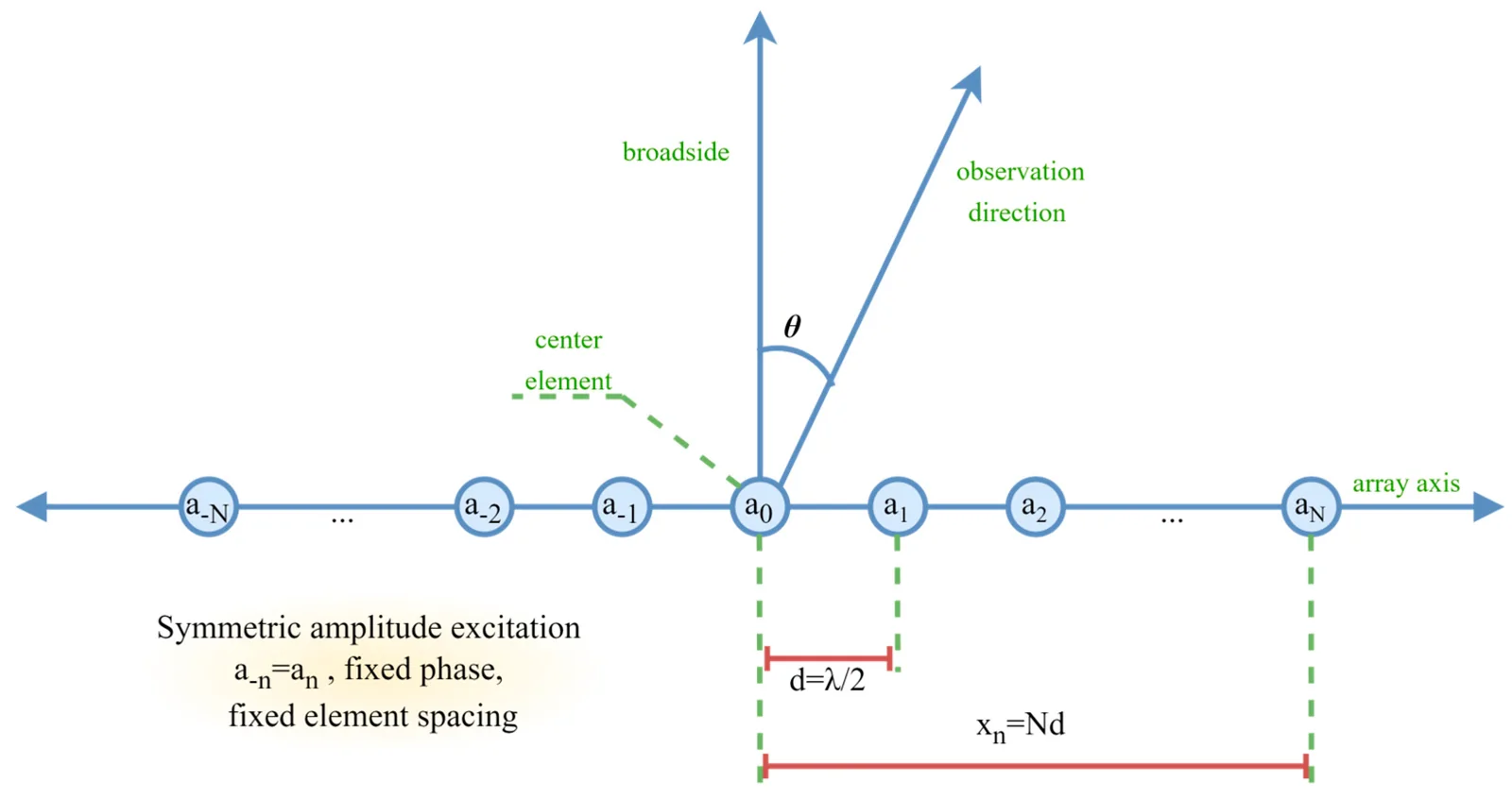
Geometry of the symmetric amplitude-controlled linear antenna array used in the array-factor formulation.

Consider a linear antenna array composed of 2N+1 isotropic radiating elements. The array is assumed to be symmetric with respect to its central element as shown in Figure 1. Hence, the central element is selected as the reference element, and the remaining elements are placed in mirrored pairs on both sides of the array center.

For a broadside linear array, the far-field radiation response can be represented by the array factor. Since the excitation phases and element locations are kept fixed in this study, the synthesis problem is reduced to the determination of real-valued amplitude coefficients. Under these assumptions, the array factor is written as follows:

(1) $$AF(\theta )=a_{0}+2\sum_{n=1}^{N}a_{n}cos(kx_{n}\sin\theta ),$$

where $a_{0}$ denotes the excitation amplitude of the central element, and $a_{n}$ represents the amplitude coefficient of the n-th element pair. The variable $x_{n}$ is the distance of the n-th element from the array center. The wavenumber is given as follows:

(2) $$k=\frac{2\pi }{\lambda }\;,$$

where λ is the wavelength. The observation angle θ is measured with respect to the broadside direction of the array.

For a uniformly spaced array, the element position can be expressed as follows:

(3) $$x_{n}=nd\;,$$

where d is the inter-element spacing. In this work, d=λ/2 is used. Therefore, grating lobes are avoided in the visible angular region, and the synthesis process focuses on side lobe control and null placement.

### 2.1. Role of Phase in Amplitude-Only Null Formation

It is important to distinguish between the phase terms involved in array pattern formation and the phase values treated as optimization variables. The term amplitude-only synthesis does not imply that phase is absent from the radiation mechanism. In (4), the angle-dependent term $kx_{n}\sin\theta_{null}$ represents the spatial phase difference produced by the element positions and observation direction. The cosine term results from combining the contributions of the symmetrically located element pairs. Thus, phase remains inherently involved in the constructive and destructive interference among the radiated fields, although the excitation phases are fixed and excluded from the decision vector.

At a prescribed interference direction $\theta_{null}$, the optimized amplitude coefficients satisfy the approximate cancellation condition:

(4) $$a_{0}+2\sum_{n=1}^{N}a_{n}\cos\left(kx_{n}{\sin\theta }_{null}\right)\approx 0\;.$$

It is targeted that SRW-PO to modify the magnitudes of the element-pair contributions so that their vector sum approaches zero at the prescribed direction. The null is produced through destructive interference governed by the spatial phase terms. Independent phase optimization is not required for this cancellation under the array geometry considered in this study. Accordingly, the excitation phases are fixed rather than physically absent.

The normalized array pattern is obtained as follows:

(5) $$AF_{n}(\theta )=\frac{|AF(\theta )|}{\underset{\theta }{max}|AF(\theta )|}\;.$$

The corresponding pattern in decibel scale is computed as follows:

(6) $$AF_{dB}(\theta )=20{log}_{10}\left(AF_{n}(\theta )+\varepsilon \right)\;,$$

where ε is a very small positive constant used to avoid numerical singularity in logarithmic evaluation.

### 2.2. Amplitude-Only Optimization Formulation

The design objective is to generate prescribed nulls at selected interference directions while keeping the synthesized pattern close to the reference Chebyshev pattern. Let $AF_{des}(\theta )$ denote the desired radiation response and $AF_{syn}(\theta )$ denote the pattern produced by a candidate amplitude vector.

(7) $$a=[a_{0},a_{1},\ldots ,a_{N}{]}^{T}\;.$$

The amplitude-only synthesis problem is formulated as a constrained numerical minimization problem. Among the established array-synthesis formulations, the present study deliberately adopts amplitude-only control. Accordingly, the excitation amplitudes constitute the optimization variables, whereas the excitation phases and element positions remain fixed. Fixing these quantities reduces the dimensionality of the search space and avoids the requirement for independently adjustable phase shifters. It does not remove the spatial phase terms from the array factor.

(8) $$\underset{a}{min}\;F(a)\;\mathrm{subject}\;\mathrm{to}{\;a}_{n}^{min}\leq a_{n}\leq a_{n}^{max},n=0,1,\ldots ,N\;.$$

The cost function includes three main design terms: null depth error, maximum side lobe error, and dynamic range ratio. It is defined as follows:

(9) $$F(a)=w_{1}E_{NDL}+w_{2}E_{MSL}+w_{3}E_{DRR}\;,$$

where $w_{1}$, $w_{2}$, and $w_{3}$ are weighting coefficients. These coefficients determine the relative importance of null suppression, side lobe limitation, and excitation realizability.

The null depth error is evaluated at the prescribed nulling angles. If the set of null directions is denoted by the following:

(10) $$\Theta_{null}=\{\theta_{1},\theta_{2},\ldots ,\theta_{Q}\},$$

then the nulling error can be defined as follows:

(11) $$E_{NDL}=\sum_{q=1}^{Q}max{\left[0,AF_{dB}(\theta_{q})-NDL_{target}\right]}^{2}.$$

Here, $NDL_{target}$ is the required null depth level in decibels. This formulation penalizes only the cases where the achieved null is shallower than the desired level. If the null depth is already lower than the target value, no additional penalty is imposed.

The side lobe control term is evaluated outside the main lobe region. Let $\Theta_{SL}$ denote the angular region excluding the main lobe. The maximum side lobe error is defined as follows:

(12) $$E_{MSL}=\underset{\theta \in \Theta_{SL}}{max}{\left[max\left(0,AF_{dB}(\theta )-MSL_{target}\right)\right]}^{2}\;,$$

where $MSL_{target}$ is the prescribed maximum side lobe level. This term prevents undesired side lobe growth during the nulling process.

The excitation dynamic range is measured by the dynamic range ratio as follows:

(13) $$DRR=\frac{max(a_{n})}{min(a_{n})},n=0,1,\ldots ,N\;.$$

To include this quantity in the objective function, the following penalty term is used:

(14) $$E_{DRR}=max{\left[0,DRR-DRR_{target}\right]}^{2}.$$

A lower DRR indicates a more balanced amplitude distribution. This is desirable for practical attenuator-based implementation because excessive amplitude variation may increase hardware complexity.

The resulting optimization problem can therefore be expressed compactly as,

(15) $$a^{*}=arg\;\underset{a}{min}\left\{w_{1}E_{NDL}+w_{2}E_{MSL}+w_{3}E_{DRR}\right\}\;.$$

The optimized vector $a^{*}$ is used to form the final radiation pattern. Since the array is symmetric, only N+1 independent amplitude values are required for a 2N+1 element array. This reduces the number of optimization variables and preserves pattern symmetry around the broadside direction.

In this formulation, the element pattern and mutual coupling effects are not included. Hence, the synthesis is carried out at the array-factor level. This assumption provides a clear mathematical model for amplitude-only null steering. However, for practical antenna implementation, embedded element responses, coupling matrices, amplitude quantization, and manufacturing tolerances can be incorporated into the same objective function framework.

### 2.3. Prescribed-Angle Deep-Null Construction Procedure

**Figure 2:**
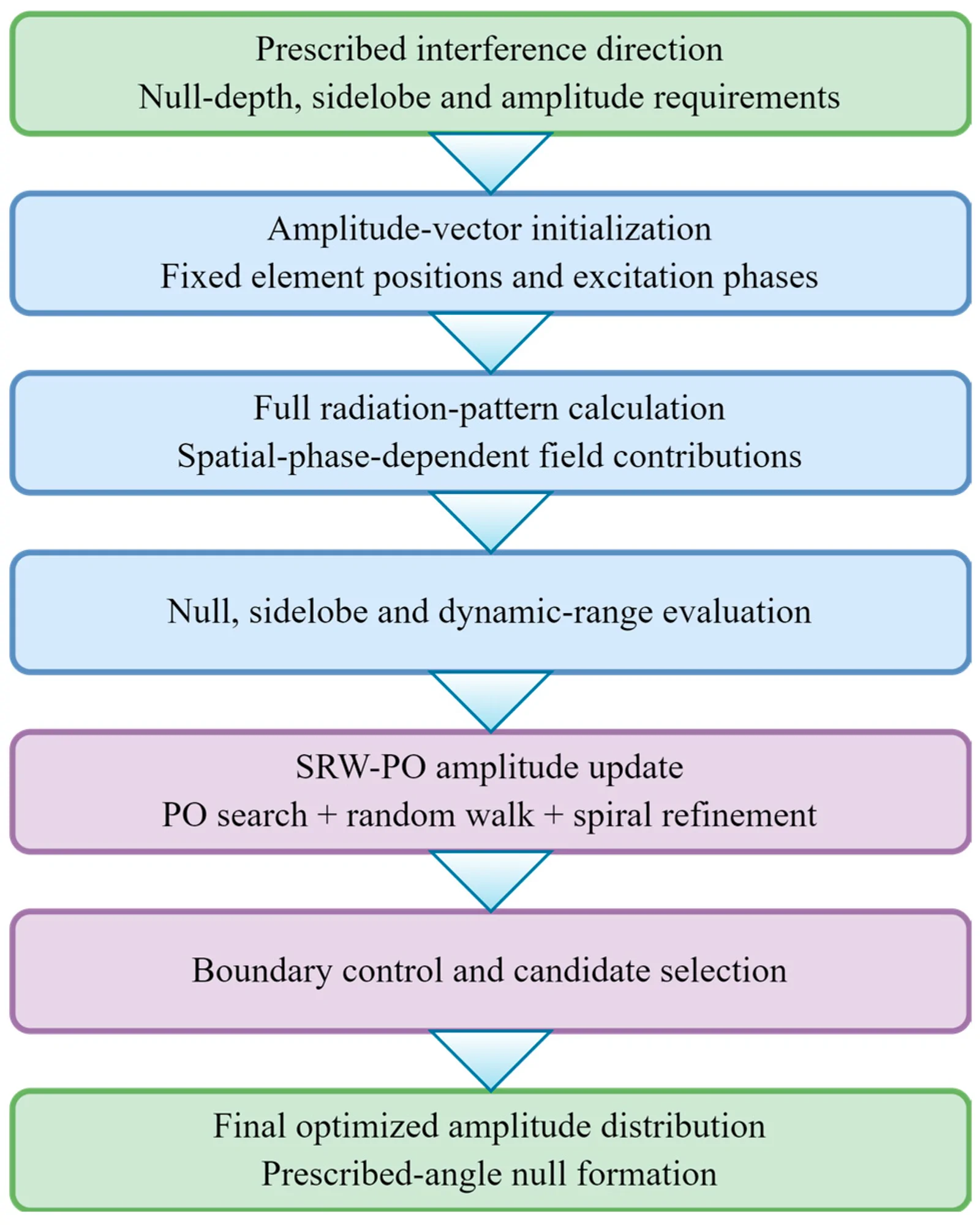
General procedure for prescribed-angle deep-null construction through fixed-phase amplitude optimization.

The proposed synthesis process begins by defining the interference direction, required null depth threshold, side lobe requirement, excitation limits, and dynamic-range constraint. The prescribed null angle is an external design input rather than a direction autonomously selected by SRW-PO. Element positions and excitation phases remain fixed. Only the independent excitation amplitudes are included in the optimization vector. The complete prescribed-angle null-synthesis procedure, from the definition of the design requirements to the generation of the optimized excitation distribution, is summarized in Figure 2.

Fixed excitation phases do not eliminate phase differences from the radiation mechanism. At an off-broadside direction, the fields radiated by different elements travel unequal distances and therefore arrive with different spatial phases. These direction-dependent phase relationships are established by the array geometry. SRW-PO modifies the strengths of these contributions until the opposing field components become balanced and the residual field at the prescribed direction is minimized.

Each candidate’s amplitude distribution generates a complete radiation pattern. Its quality is assessed according to the response at the prescribed null direction, the maximum side lobe level, and the excitation dynamic range. The adaptive random walk mechanism explores alternative amplitude balances when the search becomes unproductive. The spiral operator refines promising distributions to reduce the remaining field mismatch. Boundary control maintains feasible excitation values, while greedy selection retains the better candidate.

Changing the required null angle does not require modification of the SRW-PO operators. The new interference direction is supplied to the same pattern evaluation and objective assessment procedure. A new amplitude distribution is then optimized because each angular direction produces a different spatial-phase relationship across the array. The same mechanism can also process multiple prescribed directions by evaluating all required null locations for every candidate distribution.

## 3. Spiral Random Walk Enhanced Puma Optimizer (SRW-PO)

The proposed method is developed by preserving the main search philosophy of the classical PO and embedding two complementary search mechanisms into its update cycle. The classical PO models the hunting behavior of pumas through two main tendencies: wide range search for locating promising regions and ambush-oriented exploitation for approaching high-quality solutions. In the proposed approach, this original exploration-exploitation structure is retained, while its local refinement ability and escape capability are strengthened through spiral motion and adaptive random walk.

The main idea behind the proposed method is not to replace the original PO, but to reinforce its search dynamics with lightweight auxiliary operators. The standard puma update first guides each search agent according to the current population state, randomly selected peers, elite information, and the best solution obtained so far. After this main update, selected weak individuals are further processed by the embedded operators. This design allows the algorithm to improve poorly performing agents without disturbing the entire population structure.

The spiral motion operator is introduced to improve local search around the current best solution. During the middle and later stages of the search, weak individuals are repositioned around the best solution by a spiral trajectory. This mechanism enables the algorithm to scan the neighborhood of the best candidate from different directions instead of moving only through direct attraction. As a result, exploitation becomes more diverse and the probability of refining promising regions is increased.

The adaptive random walk operator is used to maintain diversity and reduce premature convergence. It is mainly activated in the early search stage and when stagnation is detected. The operator applies controlled perturbations to weak individuals, where the step size decreases as the iteration progresses. Therefore, the algorithm performs wider exploratory moves at the beginning and more conservative corrections near the end of the search. When stagnation occurs, the random walk scale is temporarily increased to help the population escape from local optima.

Overall, the proposed method can be viewed as a hybrid enhancement of the classical PO. The original hunting and ambush behaviors remain the backbone of the algorithm, while spiral motion strengthens exploitation, whereas adaptive random walk improves diversity and local optimum avoidance. This embedded design is particularly suitable for array antenna design problems, where the optimizer must simultaneously suppress side lobes, control nulls, and avoid poor local solutions caused by highly nonlinear radiation pattern objectives.

Let $X_{i}^{t}$ denote the i-th candidate solution at iteration t, $X_{best}^{t}$ the best solution, $X_{peer}^{t}$ a randomly selected solution, and $X_{elite}^{t}$ the mean position of the elite individuals. The normalized search progress is defined as follows:

(16) $$p=\frac{t}{T-1}\;.$$

The elite mean is computed as follows:

(17) $$X_{elite}^{t}=\frac{1}{N_{e}}\sum_{j=1}^{N_{e}}X_{j}^{t}\;.$$

At each iteration, the basic puma update switches between exploration and exploitation. The exploration candidate is generated as follows:

(18) $$X_{exp}^{t}=0.65\left[X_{i}^{t}+r_{1}\left(X_{peer}^{t}-X_{i}^{t}\right)+0.10\left(1-p\right)\left(UB-LB\right)⊙N\left(0,1\right)\right]+0.35\left[LB+r_{2}⊙\left(UB-LB\right)\right]\;.$$

The exploitation candidate is generated as follows:

(19) $$X_{exc}^{t}=X_{best}^{t}+r_{3}⊙\left(X_{elite}^{t}-X_{i}^{t}\right)+\left(2r_{4}-1\right)\left(1-p\right)⊙\left|X_{best}^{t}-X_{i}^{t}\right|\;.$$

The candidate solution is then selected as follows:

(20) $$X_{new}^{t}=\left\{\begin{array}{cc} X_{exp}^{t}, & r<1-0.55p\;,\\ X_{exc}^{t}, & \mathrm{otherwise}\;. \end{array}\right.$$

To enhance diversity, an adaptive random walk operator is embedded into weak individuals:

(21) $$X_{rw}^{t}=X_{i}^{t}+\alpha_{t}\left(UB-LB\right)⊙N\left(0,1\right)\;,$$

where

(22) $$\alpha_{t}=0.18\left(1-p\right)+0.01.$$

When stagnation occurs, the step size is enlarged as follows:

(23) $$\alpha_{t}=\alpha_{t}\min\left(2.5,\;1+\frac{s}{N}\right)\;,$$

where s is the stagnation counter. In addition, a spiral local search is applied around the best solution:

(24) $$X_{sp}^{t}=X_{best}^{t}+e^{bl}\cos\left(2\pi l\right)⊙\left|X_{best}^{t}-X_{i}^{t}\right|\;,$$

where

(25) $$b=0.95-0.55p,\;\;\;\;\;\;l∼U\left(-1,1\right)\;.$$

Equation (24) is an auxiliary local-search operator added to the original PO framework [27]. The logarithmic-spiral form [19,30,31] was selected because it provides gradient-free, distance-scaled refinement around the current best solution with low computational cost. It is mainly suitable for continuous bounded problems. Its search movement decreases as an individual approaches the best solution, while out-of-bound candidates are repaired using Equation (26). Gaussian perturbation, Lévy flight, differential mutation, and coordinate search are possible alternatives. In the present implementation, $l∼U\left({\left[-1,1\right]}^{D}\right)$ is generated component wise, and the adaptive random walk complements the spiral operator when additional diversity is required.

All generated candidates are restricted to the search bounds:

(26) $$X^{t}=\min\left(\max\left(X^{t},LB\right),UB\right)\;.$$

Finally, greedy selection is used as follows:

(27) $$X_{i}^{t+1}=\left\{\begin{array}{cc} X_{cand}^{t}, & f\left(X_{cand}^{t}\right)<f\left(X_{i}^{t}\right)\\ X_{i}^{t}, & \mathrm{otherwise}. \end{array}\;\;.\right.$$

Here, $X_{cand}^{t}$ can be a basic puma candidate, a random walk candidate, or a spiral candidate, respectively.

The proposed method can be viewed as a hybrid enhancement of the classical PO. The original hunting and ambush behaviors remain the backbone of the algorithm, while spiral motion strengthens exploitation and adaptive random walk improves diversity and local-optimum avoidance. This embedded design is particularly suitable for array antenna design problems, where the optimizer must simultaneously suppress side lobes, control nulls, and avoid poor local solutions caused by highly nonlinear radiation-pattern objectives. The reason this combination is effective is that array antenna synthesis rarely behaves like a smooth single basin optimization problem. Small changes in excitation amplitudes can reshape side lobes and null depths in a highly coupled manner, and a solution that looks promising in one angular region may still fail in another. The classical puma update provides a useful global search backbone, but the added random walk operator gives weak candidates a controlled chance to leave unproductive regions, while the spiral operator lets the search examine the neighborhood of the current best solution from multiple directions. In this way, the algorithm does not simply rush toward the best individual; it continues to test the surrounding pattern landscape before committing. This balance between correction, escape, and focused refinement is the main reason why the proposed approach is expected to produce more stable side lobe suppression and null control in array antenna design. Detailed pseudo-algorithm can be found in Algorithm 1.
**Algorithm 1.** Pseudo code of SRW-POInput: $f\left(x\right),LB,UB,N,T$
Output: $X_{best}$, $f_{best}$ 1.    Initialize $X_{i}$, i=1,…,N, within $\left[LB,UB\right]$2.    Evaluate all individuals and determine $X_{best}$3.    Set stagnation counter s=0 4.    for t=1 to T do5.            Compute progress ratio p using Equation (16)6.            Compute elite mean $X_{elite}$ using Equation (17) 7.            for i=1 to N do8.                    Select a random peer $X_{peer}$9.                    Generate the exploration candidate using Equation (18)10.                  Generate the exploitation candidate using Equation (19)11.                  Select the basic puma candidate using Equation (20)12.                  Apply boundary control using Equation (26)13.                  Update $X_{i}$ by greedy selection using Equation (27)14.              end for 15.              Update $X_{best}$ and $f_{best}$16.              Update stagnation counter s 17.              if p<0.68 or s>0 then18.                  Select weak individuals19.                  Generate random walk candidates using Equations (21)–(23)20.                  Apply boundary control using Equation (26)21.                  Accept improved candidates using Equation (27)22.              end if23.              if p>0.18 then24.                  Select weak individuals25.                  Generate spiral candidates using Equations (24) and (25)26.                  Apply boundary control using Equation (26)27.                  Accept improved candidates using Equation (27)28.              end if 29.              Update $X_{best}$ and $f_{best}$30. end for 31. return $X_{best}$, $f_{best}$

Using the exploration–exploitation terminology of the original PO, the dominant computational cost arises from updating and evaluating $N_{p}$ candidate solutions with dimension D over T iterations. If $C_{f}$ denotes the cost of one objective function evaluation, the baseline PO complexity is $O\left(TN_{p}\left(D+C_{f}\right)\right)$, with a memory requirement of $O\left(N_{p}D\right)$. SRW-PO preserves this backbone and applies random walk and spiral refinement only to fixed fractions of the population during selected iterations. Consequently, these embedded operators increase the number of evaluations but do not change the asymptotic complexity, which remains $O\left(TN_{p}\left(D+C_{f}\right)\right)$ in time and $O\left(N_{p}D\right)$ in memory.

## 4. Numerical Examples and Results

### 4.1. Experimental Setup and Benchmark Configuration

This section defines the general experimental framework used to evaluate the optimization performance of the proposed SRW-PO algorithm. The benchmark problem is formulated under a fixed antenna array synthesis scenario, where all candidate algorithms are tested using the same array geometry, objective function, variable boundaries, stopping criteria, and computational budget. This common configuration is essential to ensure that the observed performance differences arise from the search mechanisms of the algorithms rather than from inconsistent simulation conditions or unequal parameter settings.

The comparative study includes five population-based metaheuristic algorithms: the proposed SRW-PO, the original PO, genetic algorithm (GA), whale optimization algorithm (WOA), and grey wolf optimizer (GWO). For each algorithm, the population size is set to 30, and the maximum number of iterations is fixed at 1000. These values provide a balanced search budget by allowing sufficient population diversity during exploration while maintaining a consistent number of function evaluations across all methods. Therefore, each algorithm is given the same opportunity to investigate the solution space and refine candidate excitation configurations.

In (26), LB and UB denote the lower and upper-bound vectors of the optimization variables, respectively. For the present antenna synthesis problem, every component of these vectors is set to $LB_{n}=-0.20$ and $UB_{n}=+0.20$. The 25-element symmetric array is represented by 13 independent decision variables, where each variable is an additive amplitude correction applied to the corresponding initial −25 dB Chebyshev coefficient. Accordingly, the physical excitation coefficient is calculated as $a_{n}=a_{n}^{Cheb}+X_{n}$, with $-0.20\leq X_{n}\leq +0.20$. The excitation phases and the 0.5λ element spacing remain fixed. Any candidate component below $LB_{n}$ is replaced by −0.20, whereas any component above $UB_{n}$ is replaced by +0.20 before objective evaluation. The radiation pattern is evaluated over $\left[-90^{\circ },90^{\circ }\right]$ with a $0.25^{\circ }$ angular resolution, while the null directions and required null depths are defined separately for each synthesis scenario.

Since metaheuristic algorithms are stochastic by nature, their performance cannot be reliably assessed from a single execution. To obtain statistically meaningful results, each algorithm is independently executed 100 times under the same benchmark configuration. The independent runs make it possible to evaluate not only the best achieved solution but also the average convergence behavior, consistency, and repeatability of each method. This experimental setup provides a fair and technically consistent basis for comparing SRW-PO against both its baseline version and widely used evolutionary and swarm intelligence optimizers.

The null directions considered in all subsections of Section 4 were deliberately prescribed rather than randomly selected or generated by the SRW-PO algorithm. Specifically, 15.50°, 25.25°, and 36.00° correspond to distinct and well-separated side lobe peak regions of the initial −25 dB Chebyshev pattern. These directions were selected to construct nontrivial single and multiple-nulling scenarios in which initially significant side lobe radiation must be suppressed, while avoiding direct modification of the main beam region. In each scenario, the selected angles are fixed inputs to the optimization process; SRW-PO optimizes only the element amplitudes required to produce destructive field superposition in these prescribed directions. Therefore, the reported null depths are optimization outcomes rather than predefined depth values. The same formulation can accept different prescribed null directions without modifying the optimization mechanism, although the attainable null depth and pattern trade-offs depend on the location, number, and angular separation of the requested nulls.

To quantify the effect of amplitude weighting on the broadside gain, an array factor-based gain metric is evaluated under equal total excitation power as follows:

(28) $$G_{array}=\frac{{\left|\sum_{n=1}^{N}a_{n}\right|}^{2}}{\sum_{n=1}^{N}{\left|a_{n}\right|}^{2}}\;,$$

where $a_{n}$ denotes the physical excitation amplitude of the n-th element in the complete 25-element array, including the symmetrically mirrored elements. The numerator represents the squared coherent field contribution in the broadside direction, whereas the denominator represents the total excitation power. Therefore, $G_{array}$ measures the broadside array gain reduction caused by amplitude redistribution. The corresponding gain loss is calculated relative to the initial Chebyshev distribution as,

(29) $$L_{G}=10\log_{10}\left(G_{array,ref}/G_{array,opt}\right)\;.$$

This array factor-based metric assumes identical lossless elements and is used to isolate the effect of amplitude weighting from element gain, feeding losses, and mutual coupling.

### 4.2. Baseline Chebyshev Pattern

**Figure 3:**
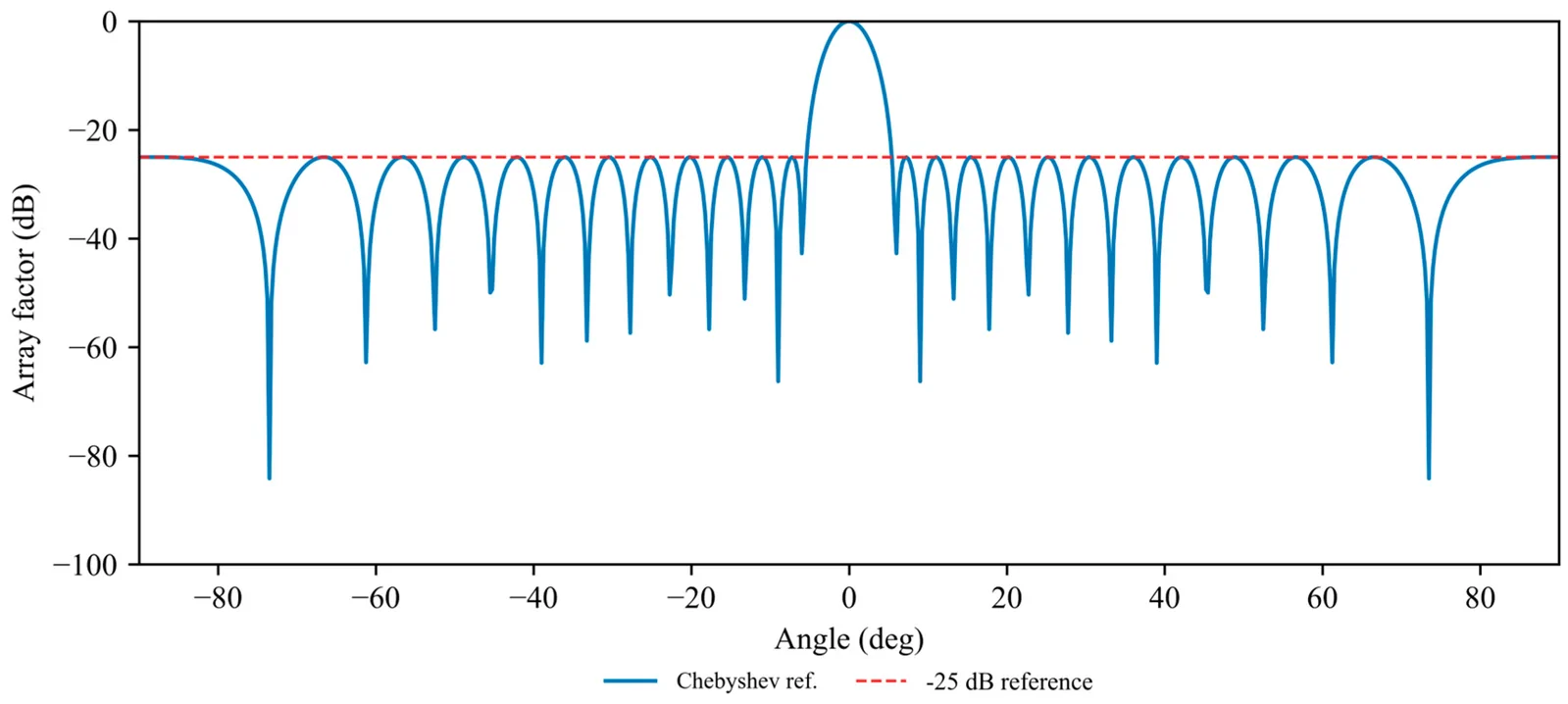
Initial radiation pattern generated using the −25 dB Dolph–Chebyshev excitation distribution [4]. The red dashed line indicates the −25 dB side lobe reference.

Figure 3 presents the initial radiation pattern obtained from the Chebyshev-based excitation distribution before applying any metaheuristic optimization procedure. This pattern is used as the reference starting point of the synthesis process and provides a controlled baseline for evaluating the improvement capability of the optimization algorithms. The dashed reference line at −25 dB indicates the target side lobe level considered in this study, allowing the initial array response to be visually assessed with respect to the desired suppression threshold.

As observed in Figure 3, the main beam is preserved around the broadside direction, while the side lobe region remains close to the prescribed −25 dB level. The Chebyshev initialization already provides a well-structured and physically meaningful excitation profile rather than a random starting condition; however, the presence of side lobe peaks near the reference boundary also shows that further optimization is still required to improve side lobe suppression, stabilize the radiation response, and obtain a more favorable trade-off between main lobe preservation and side lobe reduction.

### 4.3. Single-Null Synthesis Performance

**Figure 4:**
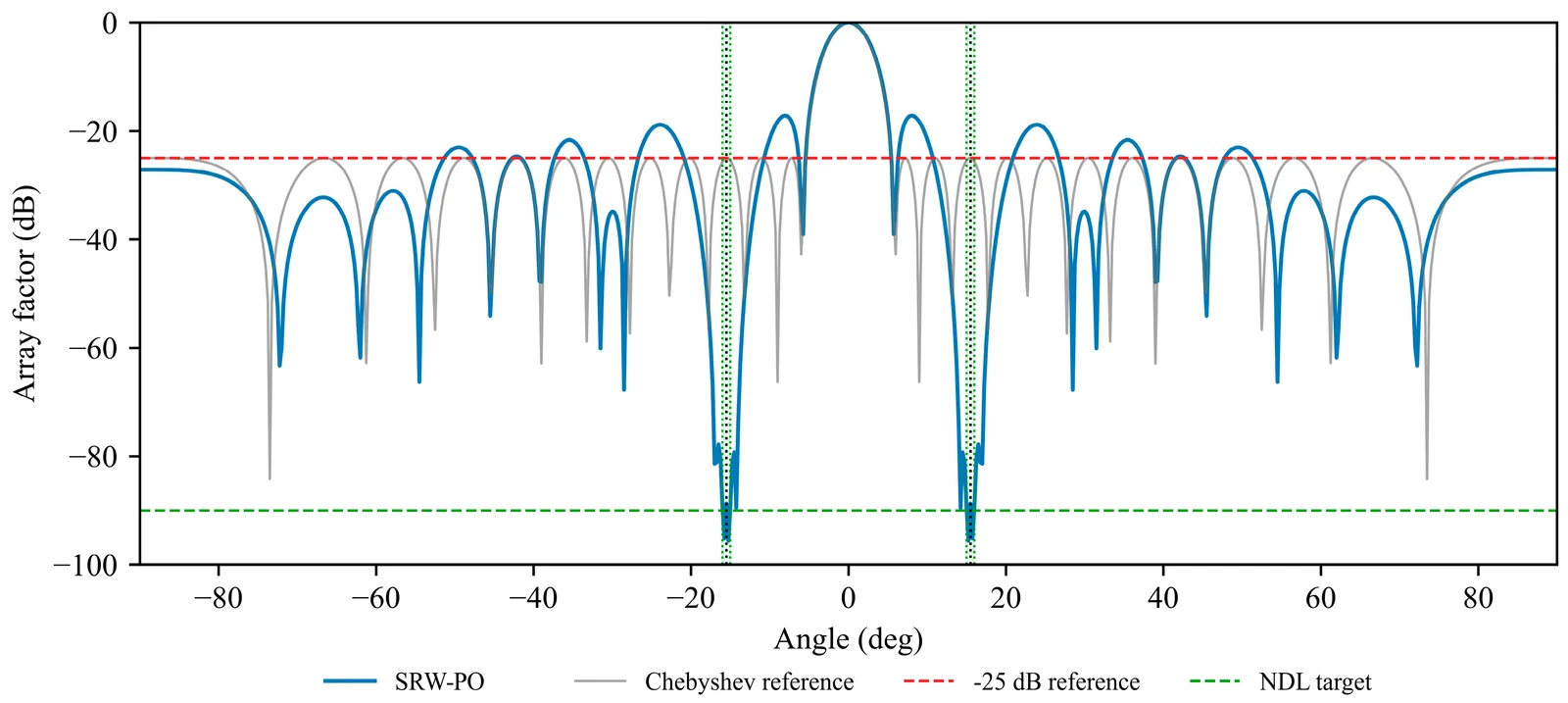
Single-null radiation pattern optimized by SRW-PO around 15.50 deg. The black dotted vertical lines indicate the prescribed null directions at ±15.50°, while the green dotted vertical lines and shaded regions define the corresponding ±0.50° null evaluation windows. The horizontal green dashed line indicates the target null depth level.

Figure 4 shows the optimized radiation pattern for the single-null case, where the objective is to place a deep attenuation point around 15.50 deg while keeping the main beam at broadside. The grey curve corresponds to the Chebyshev reference pattern, and the blue curve represents the SRW-PO-optimized response. The red dashed line indicates the −25 dB side lobe reference, whereas the green dashed line marks the target null depth level. This experiment is included to examine how effectively the excitation amplitudes can be adjusted to suppress the array response in a prescribed interference direction.

The resulting pattern shows that SRW-PO forms a deep numerical null in the target angular region. Based on the extracted metrics, the representative single-null result reaches a null depth of approximately −95.579 dB at 15.50 deg. The maximum side lobe level is −17.1655 dB, while the HPBW and MLW are 4.75 deg and 11.5 deg, respectively. Compared with the Chebyshev reference, which provides a regular side lobe structure near the −25 dB level but does not impose a dedicated null at the selected direction, the optimized pattern introduces strong directional suppression at the cost of a moderate side lobe-level relaxation. The −25 dB dashed line is retained as the Chebyshev reference level and is not treated as a hard feasibility boundary in all synthesis cases.

**Figure 5:**
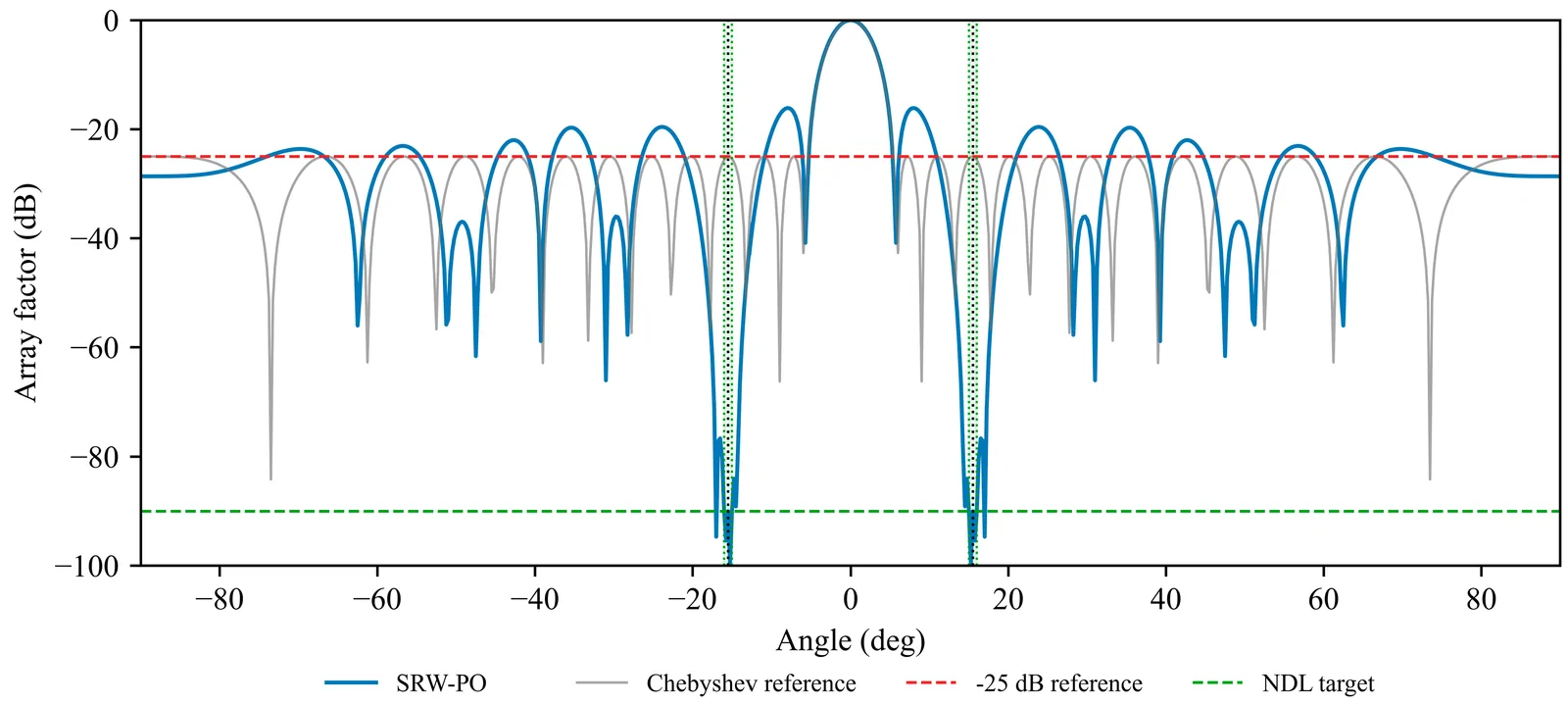
Single-null radiation pattern with dynamic range ratio control. The black dotted vertical lines indicate the prescribed null directions at ±15.50°, while the green dotted vertical lines and shaded regions define the corresponding ±0.50° null-evaluation windows. The horizontal green dashed line indicates the target null depth level.

Figure 5 presents the single-null synthesis result when dynamic range ratio (DRR) control is included in the optimization process. The purpose of this case is not only to place a deep null around the prescribed interference direction, but also to constrain the excitation amplitude variation so that the resulting array weights remain more suitable for practical implementation. As in the previous case, the Chebyshev reference pattern is shown in grey, the SRW-PO-optimized pattern is shown in blue, the red dashed line represents the −25 dB side lobe reference, and the green dashed line denotes the null depth target.

According to the extracted metrics, the optimized pattern reaches a null depth of approximately −99.580 dB at 15.50 deg. The reported HPBW is 4.75 deg, and the MLW is 11.5 deg, showing that the main beam width is preserved while the null constraint is satisfied. The DRR value is approximately 7.6626, indicating the excitation amplitude range associated with the DRR-controlled solution. The maximum side lobe level is reported as −16.1141 dB, which reflects the trade-off introduced by simultaneous null placement and dynamic range consideration.

**Figure 6:**
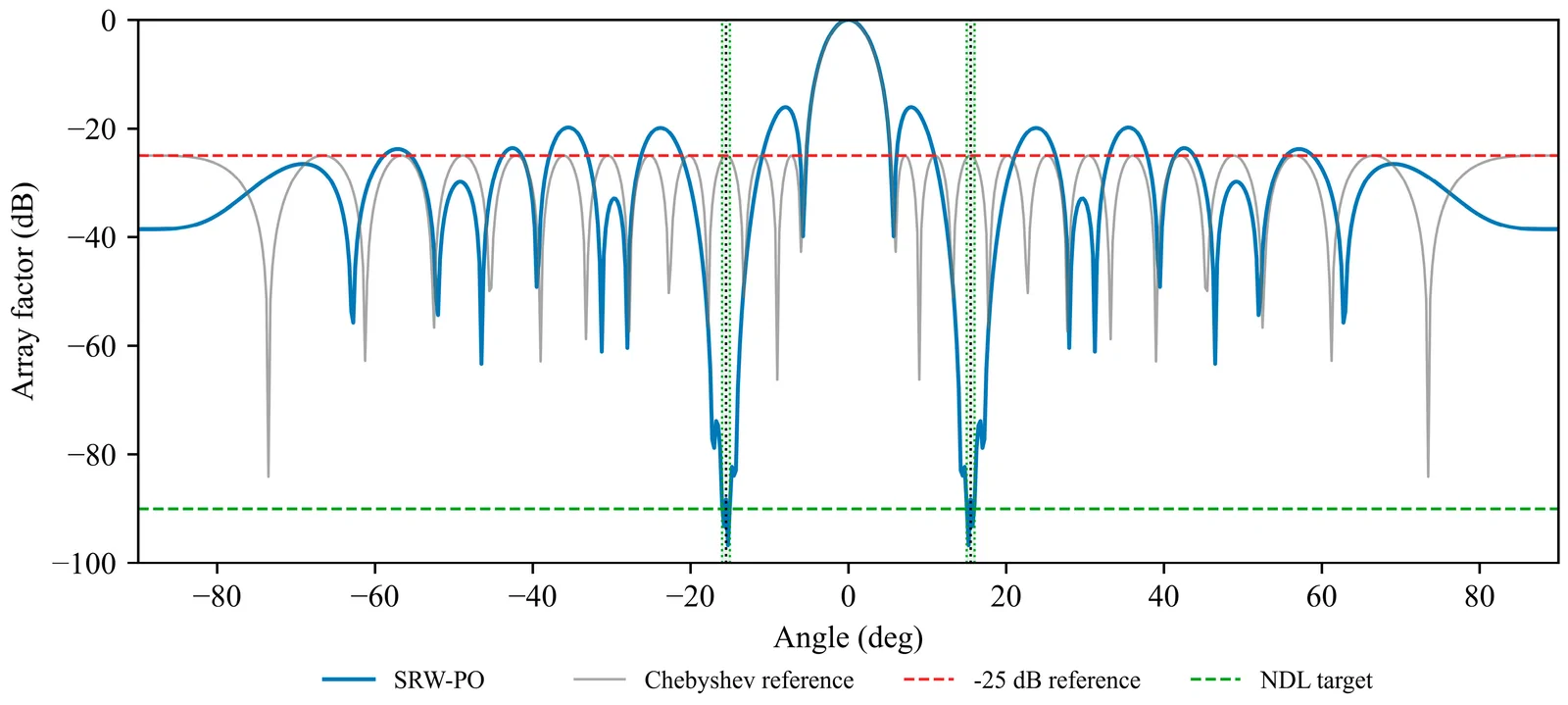
Single-null radiation pattern under a stricter maximum side lobe level condition. The black dotted vertical lines indicate the prescribed null directions at ±15.50°, while the green dotted vertical lines and shaded regions define the corresponding ±0.50° null evaluation windows. The horizontal green dashed line indicates the target null depth level.

Figure 6 shows the single-null case under a stricter side lobe-related objective setting, although the final MSL still reflects the null depth trade-off. In this configuration, the optimization problem includes the same prescribed null region around the interference direction, but the side lobe behavior is treated with a stronger constraint compared with the previous single-null cases. The Chebyshev reference pattern is given in grey, while the SRW-PO pattern is shown in blue. The red dashed line indicates the −25 dB reference level, and the green dashed line represents the target null depth level.

Based on the extracted metrics, the optimized pattern reaches a null depth of approximately −96.760 dB at 15.50 deg. The maximum side lobe level is −16.0781 dB, while the HPBW and MLW are 4.75 deg and 11.5 deg, respectively. These values show that the prescribed null is still formed in the target region, although the stricter side lobe-related setting changes the side lobe distribution around the main beam. The DRR value is approximately 7.1001, indicating the excitation amplitude range associated with this solution.

**Figure 7:**
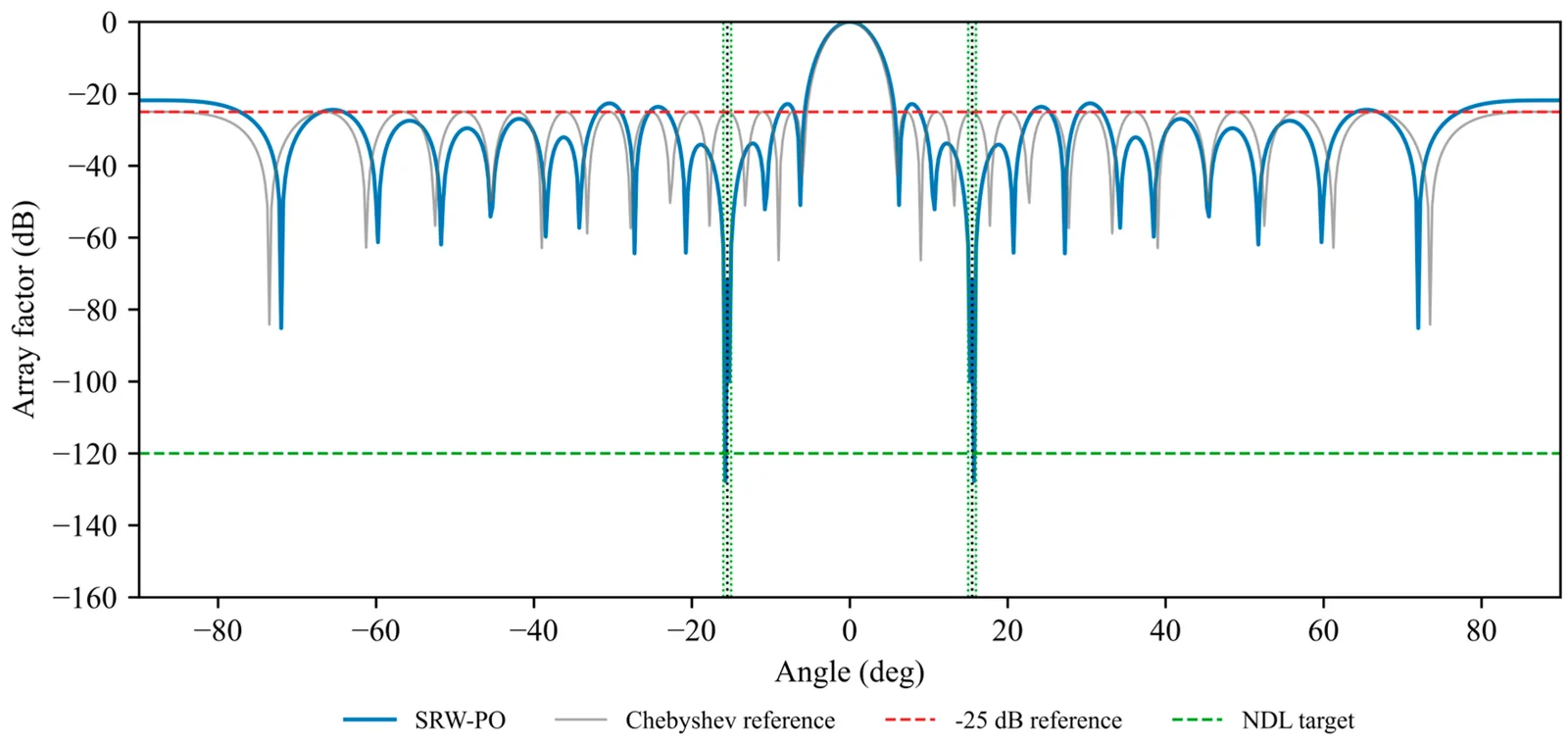
Deep single-null radiation pattern with an increased null depth requirement. The black dotted vertical lines indicate the prescribed null directions at ±15.50°, while the green dotted vertical lines and shaded regions define the corresponding ±0.50° null evaluation windows. The horizontal green dashed line indicates the target null depth level.

Figure 7 presents the deep single-null case, in which the optimization objective is configured with a more stringent null depth requirement at the prescribed interference direction. Compared with the earlier single-null cases, the target attenuation level is shifted to a lower dB value, forcing the optimizer to redistribute the excitation amplitudes more strongly toward suppressing the selected angular sector. Based on the extracted metrics, the SRW-PO pattern reaches a null depth of approximately −127.719 dB at 15.50 deg, thereby satisfying the −120 dB deep null target. The maximum side lobe level is −21.8273 dB, while the HPBW and MLW are 4.75 deg and 12.5 deg, respectively. The DRR value is approximately 5.9925, indicating the excitation amplitude range associated with this deeper spatial suppression. Overall, the result shows that SRW-PO can form a very deep numerical null while preserving the broadside main beam, although this improvement is accompanied by the expected trade-off between null depth, side lobe behavior, and excitation redistribution.

### 4.4. Multiple-Null Synthesis Performance

After evaluating the single-null configurations, the synthesis problem is extended to multiple prescribed interference directions. This step is included to examine how the optimized excitation distribution changes when more than one angular sector must be suppressed at the same time. In these cases, the objective function includes simultaneous null depth requirements while the main beam is kept at broadside, and the side lobe behavior is monitored with respect to the −25 dB reference level. Therefore, the following figures focus on the pattern-level effect of increasing the number of imposed nulls.

**Figure 8:**
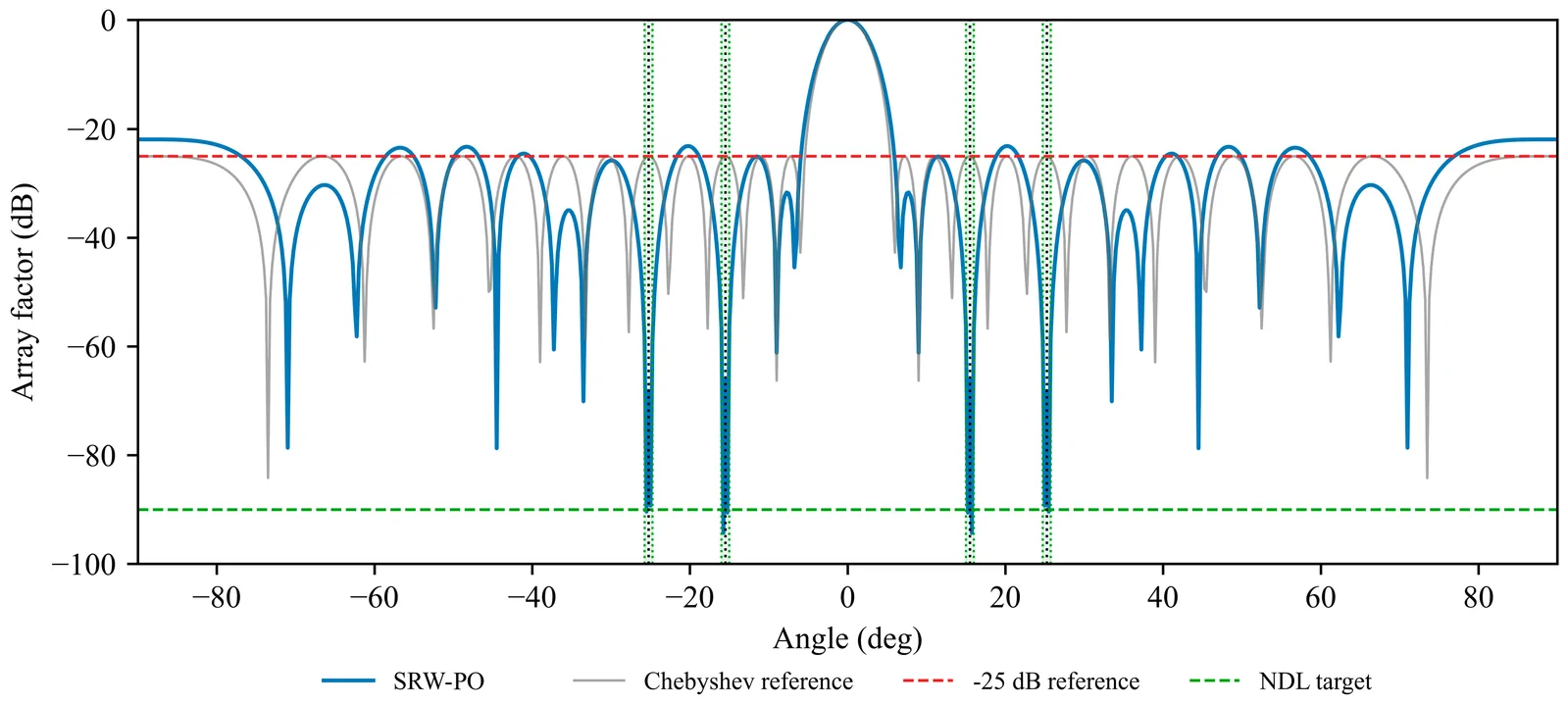
Two-null radiation pattern with prescribed nulls around 15.50 deg and 25.25 deg. The black dotted vertical lines indicate the prescribed null directions, while the green dotted vertical lines and shaded regions define the corresponding ±0.50° null evaluation windows. The horizontal green dashed line indicates the target null depth level.

Figure 8 presents the radiation pattern for the two-null case, where nulls are imposed around 15.50 deg and 25.25 deg. The optimized SRW-PO pattern forms attenuation regions at both prescribed angular locations while preserving the main beam around 0 deg. According to the extracted metrics, the achieved null depths are −94.288 dB at 15.50 deg and −90.394 dB at 25.25 deg. The maximum side lobe level is −21.9091 dB, while the HPBW and MLW are 4.75 deg and 13.5 deg, respectively. The DRR value is 5.8320, indicating the excitation amplitude range associated with this two-null solution. These results show that the excitation amplitudes are redistributed to satisfy two simultaneous spatial suppression constraints.

**Figure 9:**
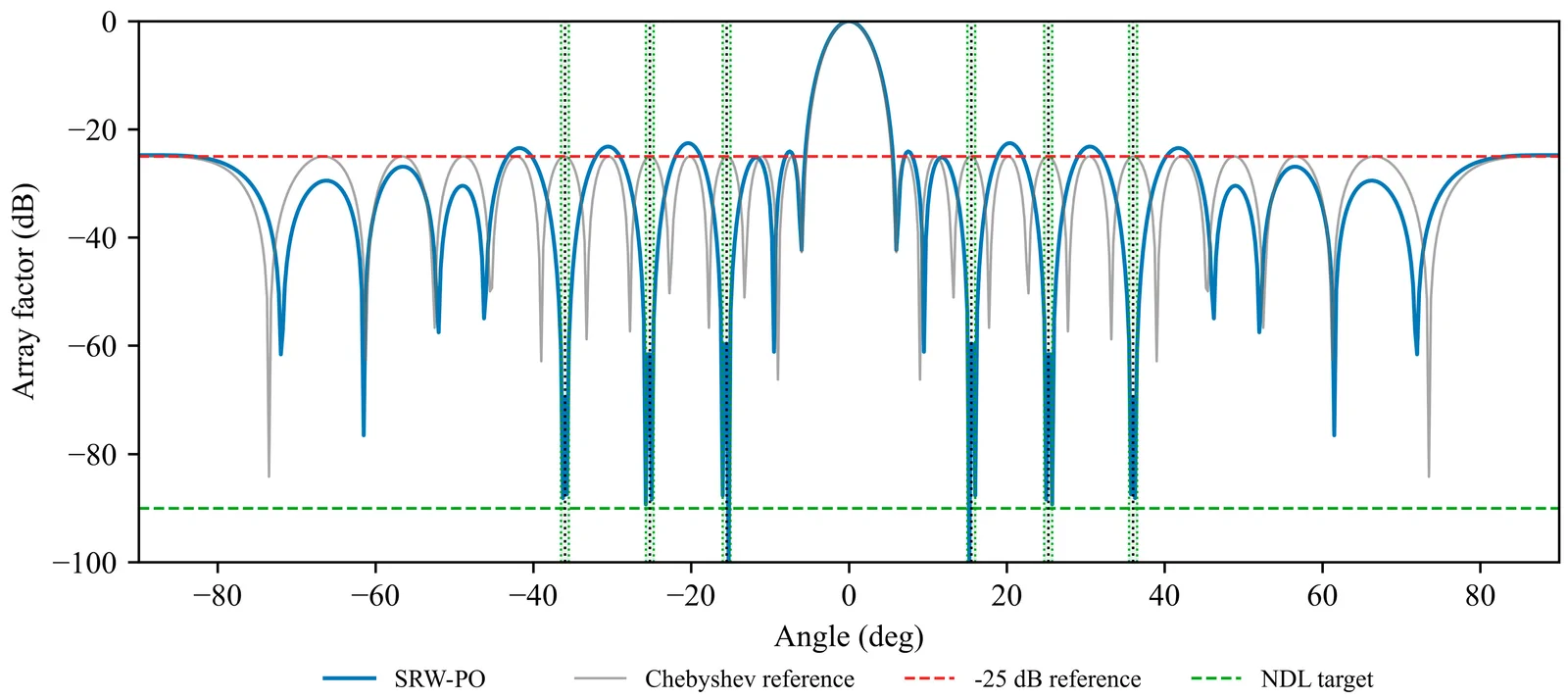
Three-null radiation pattern with prescribed nulls around 15.50 deg, 25.25 deg, and 36.00 deg. The black dotted vertical lines indicate the prescribed null directions, while the green dotted vertical lines and shaded regions define the corresponding ±0.50° null evaluation windows. The horizontal green dashed line indicates the target null depth level.

Figure 9 shows the three-null case, where the prescribed null locations are around 15.50 deg, 25.25 deg, and 36.00 deg. Compared with the two-null configuration, this case imposes an additional angular suppression requirement and therefore further constrains the available excitation degrees of freedom. According to the extracted metrics, the achieved null depths are −100.000 dB at 15.50 deg, −89.294 dB at 25.25 deg, and −88.136 dB at 36.00 deg. The maximum side lobe level is −22.5625 dB, while the HPBW and MLW are 4.75 deg and 12.0 deg, respectively. The DRR value is 3.3799, indicating a more compact excitation amplitude range compared with the previous single-null cases. The resulting pattern shows that SRW-PO can impose multiple separated nulls while preserving the broadside main beam.

### 4.5. Broad-Null Sector Synthesis

**Figure 10:**
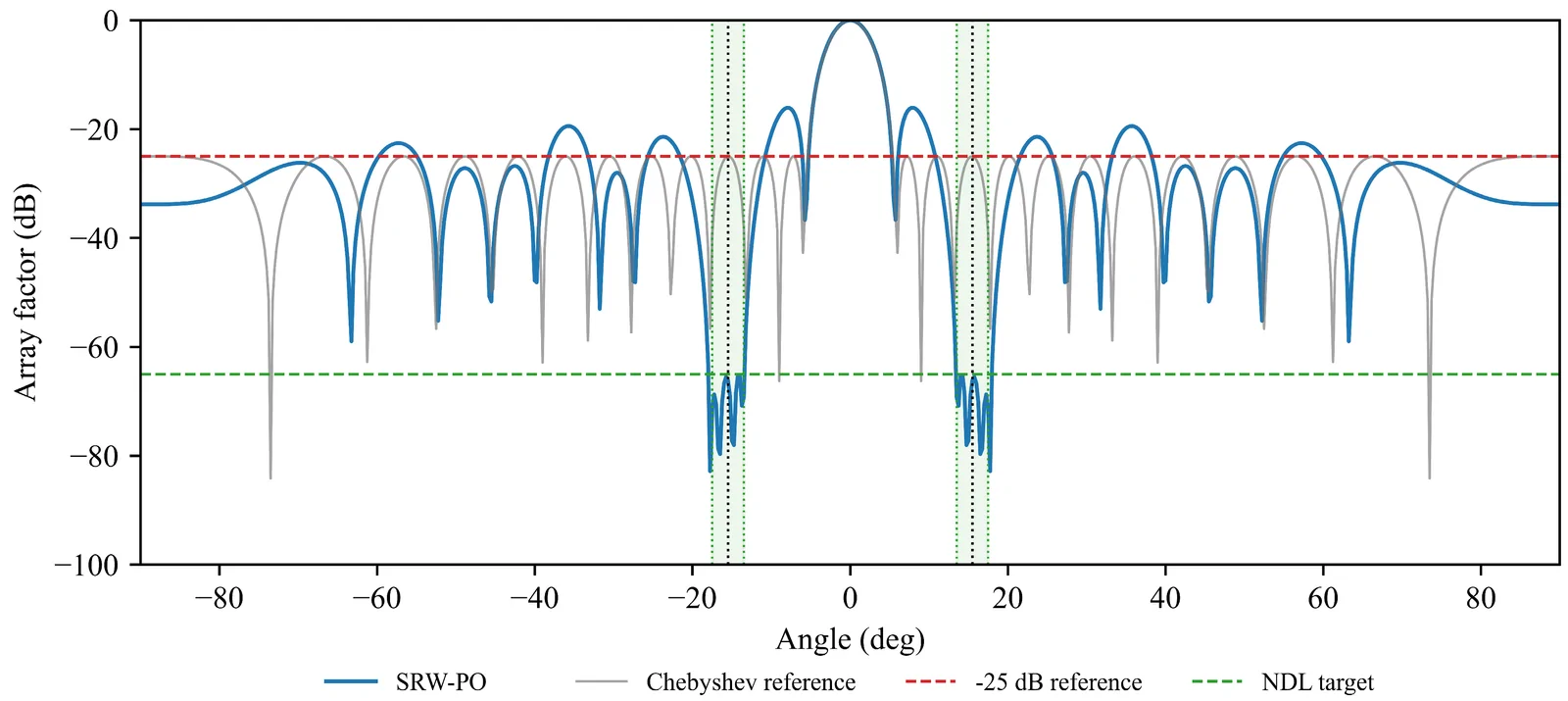
Full-angle radiation pattern for the broad-null case. The black dotted vertical lines indicate the prescribed sector centers at ±15.50°, while the green dotted vertical lines and shaded regions define the corresponding ±2.00° broad-null evaluation windows. The horizontal green dashed line indicates the −65 dB target broad-null threshold.

Figure 10 gives the full radiation pattern view over the −90 deg to 90 deg angular range for the broad-null case. This configuration is generated to evaluate the effect of suppressing a continuous angular interval rather than forcing a null at a single discrete direction. The main beam remains centered at broadside, with an HPBW of 4.75 deg and an MLW of 11.5 deg. The achieved null depth is approximately −79.703 dB at 15.50 deg, while the maximum side lobe level is −16.0791 dB. These values show that the broader suppression requirement changes the side lobe distribution compared with the narrower null cases. The DRR is approximately 6.6963, indicating the excitation amplitude range associated with this broader angular constraint.

**Figure 11:**
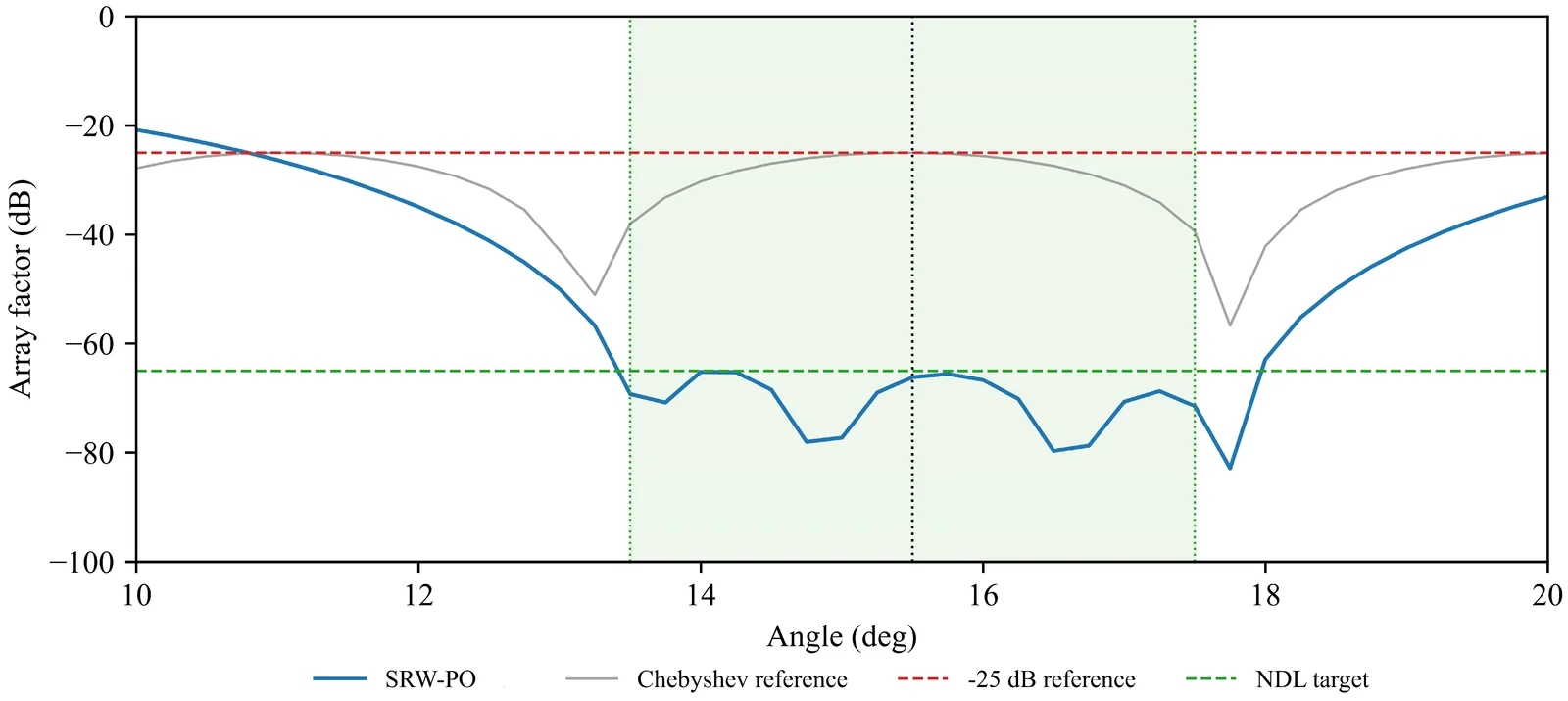
Zoomed view of the prescribed broad-null sector. The black dotted vertical line indicates the sector center at 15.50°, while the green dotted vertical lines and shaded region define the 13.50–17.50° evaluation window. The horizontal green dashed line indicates the −65 dB target broad-null threshold.

Figure 11 displays the radiation pattern over the 10–20 deg observation window. The shaded interval indicates the prescribed broad-null sector, approximately 13.5–17.5 deg. Within this sector, the maximum array factor level is −65.72 dB, which remains 0.72 dB below the −65 dB target. This confirms that the attenuation is not limited to a single angular sample; instead, the response is suppressed over the wider target interval. The zoomed plot is therefore used to verify the broad-null constraint locally, while the full angle plot is used to check the accompanying changes in the overall radiation pattern.

## 5. Results and Analysis

### 5.1. Element Excitation and Radiation Metrics

Table 1 lists the normalized excitation amplitudes used to generate the radiation patterns shown in the corresponding figures. The center element is normalized to unity, and the remaining values are given for symmetric element pairs. The Chebyshev initialization in Figure 3 has a smoother amplitude taper, whereas the optimized cases require different amplitude distributions depending on the imposed null, side lobe, and dynamic range constraints. The single-null and broad-null cases show larger variations in several symmetric element pairs, indicating that the excitation amplitudes are reshaped to satisfy angular suppression requirements.

Table 1 and Table 2 show that SRW-PO modifies the excitation distribution according to the imposed null, side lobe, and dynamic range constraints. In Table 1, the center element is normalized to 1.0000 for all cases, and the remaining amplitudes are reported for symmetric element pairs. In the Chebyshev reference case shown in Figure 3, the amplitudes decrease more smoothly from the center toward the outer elements. In contrast, the SRW-PO-based null control cases redistribute the excitation weights to satisfy the prescribed angular suppression requirements. For example, in Figure 4, the ±2 element pair reaches an amplitude of 3.8114, while the outer ±11 pair is reduced to 0.4901. In the three-null case of Figure 9, the outer excitation levels are further reduced, with the ±12, ±11, and ±10 pairs taking values of 0.7111, 0.4359, and 0.6399, respectively. This behavior indicates that the aperture energy is redistributed according to the null control and side lobe suppression requirements.

Table 2 further shows how this excitation distributions affect the radiation pattern characteristics. The HPBW remains fixed at 4.75 deg in all optimized cases, indicating that the main beam width is preserved while additional null and side lobe constraints are imposed. In the single-null cases, Figure 4 achieves a null depth of −95.579 dB at 15.50 deg, while Figure 5 and Figure 6 reach −99.580 dB and −96.760 dB, respectively, in the same direction. In the deeper null scenario, Figure 7 provides a stronger attenuation level of −127.719 dB at 15.50 deg. The method also preserves deep attenuation levels in multiple-null cases. Figure 8 obtains null depths of −94.288 dB at 15.50 deg and −90.394 dB at 25.25 deg, whereas Figure 9 achieves three nulls with depths of −100.000 dB, −89.294 dB, and −88.136 dB. At the same time, the MSL values for Figure 7, Figure 8 and Figure 9 remain at −21.8273 dB, −21.9091 dB, and −22.5625 dB, respectively. For the broad-null case in Figure 10, the response reaches a null depth of −79.703 dB at 15.50 deg, with an MSL of −16.0791 dB and a DRR of 6.6963. Although this case produces a less deep minimum attenuation than the discrete-null cases, it suppresses the response over a wider angular interval. The zoomed view in Figure 11 further confirms that the array response remains below the target NDL level across the prescribed broad-null sector rather than only at a single angular sample. These results indicate that SRW-PO can preserve the main beam while imposing deep null placement, side lobe suppression, and dynamic range control.

The effect of amplitude weighting on broadside gain was evaluated under equal total excitation power using the array factor-based gain metric. By investigating the last column of the Table 2, the initial Chebyshev distribution provides a relative broadside array gain of 13.632 dB, whereas the optimized cases range from 13.329 to 13.507 dB, corresponding to gain reductions of only 0.126–0.303 dB. The deepest null case, reaching −127.719 dB, produces a gain reduction of approximately 0.220 dB. These results are obtained using the adopted 25-element symmetric linear array and indicate that substantial interference suppression is achieved with a limited broadside-gain penalty. The positive bounded amplitudes and reported DRR values also provide a suitable basis for future implementation with practical feeding networks and hardware validation.

### 5.2. Comparative Convergence Analysis

**Figure 12:**
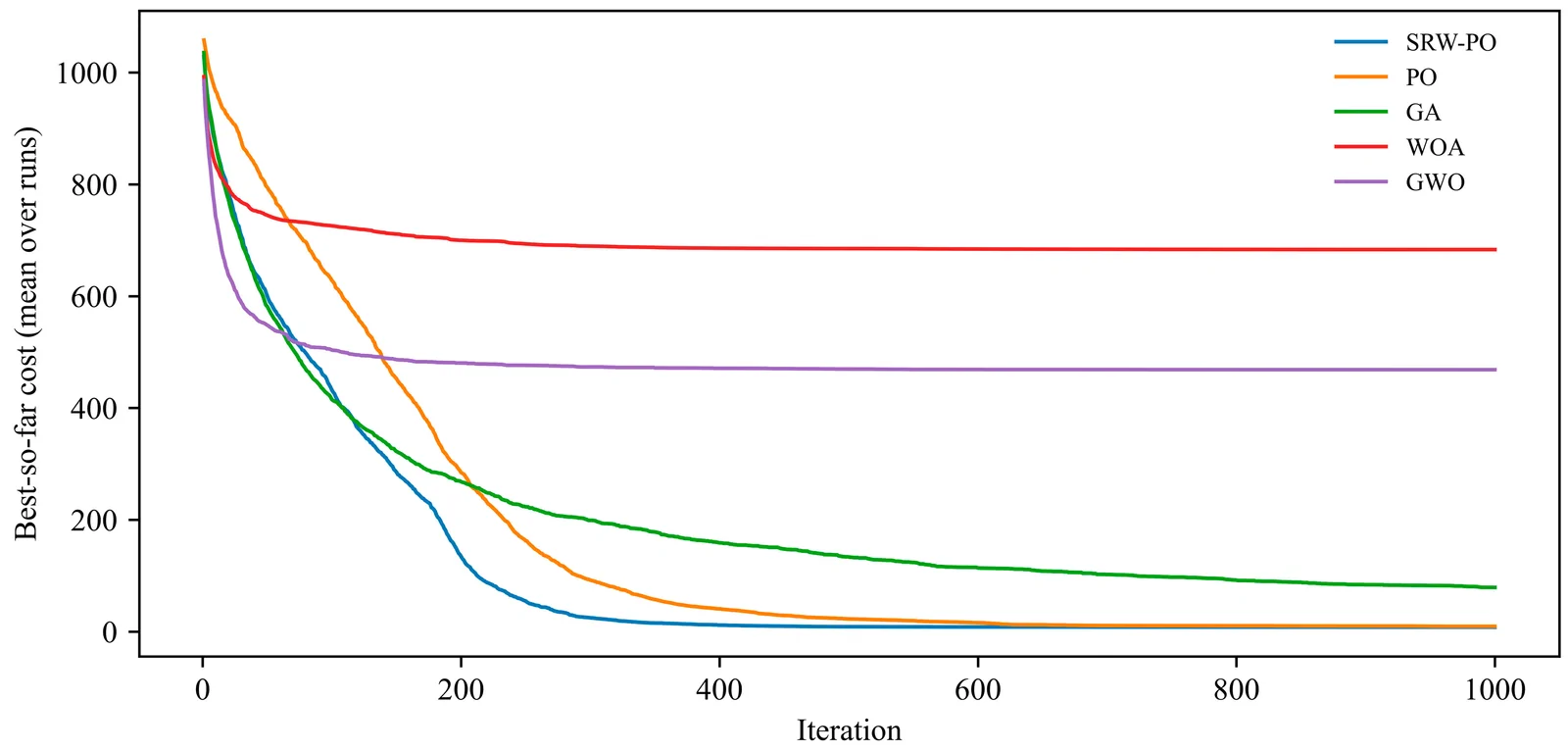
Raw comparative convergence curves of the evaluated algorithms for the broad-null benchmark.

Figure 12 shows the raw comparative convergence curves of SRW-PO, PO, GA, WOA, and GWO for the broad-null benchmark case. The curves are plotted directly in terms of the objective function value without normalization; therefore, the vertical axis preserves the actual cost scale produced by each algorithm. At the first iteration, the mean best cost values are on the order of 10^3^ for all methods, with SRW-PO starting around 1028.17, PO around 1057.90, GA around 1035.73, WOA around 992.45, and GWO around 985.22. This indicates that the initial population-level solutions are located in a similar cost range before the search process begins to separate the algorithms.

As the iterations proceed, the raw convergence curves show different reduction rates and final mean-cost levels. At the 1000th iteration, SRW-PO reaches a mean best cost of 8.01, while PO reaches 9.41. GA decreases to 79.06, whereas GWO and WOA remain at higher mean cost values of 468.45 and 683.29, respectively. The best single run costs over 100 runs are 6.89 for SRW-PO, 6.81 for PO, 7.58 for GA, 8.00 for WOA, and 264.24 for GWO. Although PO obtains a slightly lower best single run cost, SRW-PO provides the lowest mean cost and a much smaller cost dispersion. Since the raw plot keeps the original objective function scale, the algorithms with larger residual costs remain visibly separated from the lower cost solutions throughout the later iterations.

### 5.3. Statistical Benchmark Results

Table 3 shows that the comparison is conducted under the same number of independent runs, population size, and iteration limit for all algorithms. SRW-PO performs 44,449.71 function evaluations per run, whereas PO, GA, WOA, and GWO perform 30,030 evaluations. This approximately 48.0% increase is not caused by assigning an additional run or iteration budget to SRW-PO; it arises from the evaluation of spiral and random walk candidates within the standard 1000-iteration process. The corresponding mean runtime increases from 1.4918 s for PO to 2.2546 s for SRW-PO. However, the evaluation throughput remains almost unchanged, with 20,154.11 FE/s for SRW-PO and 20,141.38 FE/s for PO. Thus, the additional computational load is directly associated with the embedded operators rather than inefficient implementation. Under this setting, SRW-PO reduces the mean objective cost from 9.4067 to 8.0121 relative to PO, corresponding to an improvement of approximately 14.8%. More importantly, the standard deviation decreases from 11.1254 to 1.0771, which represents a reduction of approximately 90.3% in run-to-run dispersion. Although PO obtains a marginally lower best single run cost of 6.8113 compared with 6.8915 for SRW-PO, the proposed method produces a lower average cost and a much narrower result distribution executions.

All experiments were conducted on a 64-bit Windows computer equipped with a 13th Gen Intel Core i5-13420H processor, 12 logical processors, and 16 GB of RAM. The mean execution times measured over 100 independent runs are reported in Table 3 together with the number of function evaluations and evaluation throughput. Therefore, the comparison remains fair in terms of runs, population size, and iteration limit, while the higher function evaluation count is reported explicitly as an intrinsic computational cost of the added search operators.

The “Best” columns in Table 4, report the radiation pattern metrics obtained from the run producing the minimum composite objective cost. Therefore, these values do not necessarily correspond to the independently best MSLL, minimum NDL, or maximum NDL values since the objective function jointly considers side lobe level, null depth characteristics, HPBW, and DRR.

**Figure 13:**
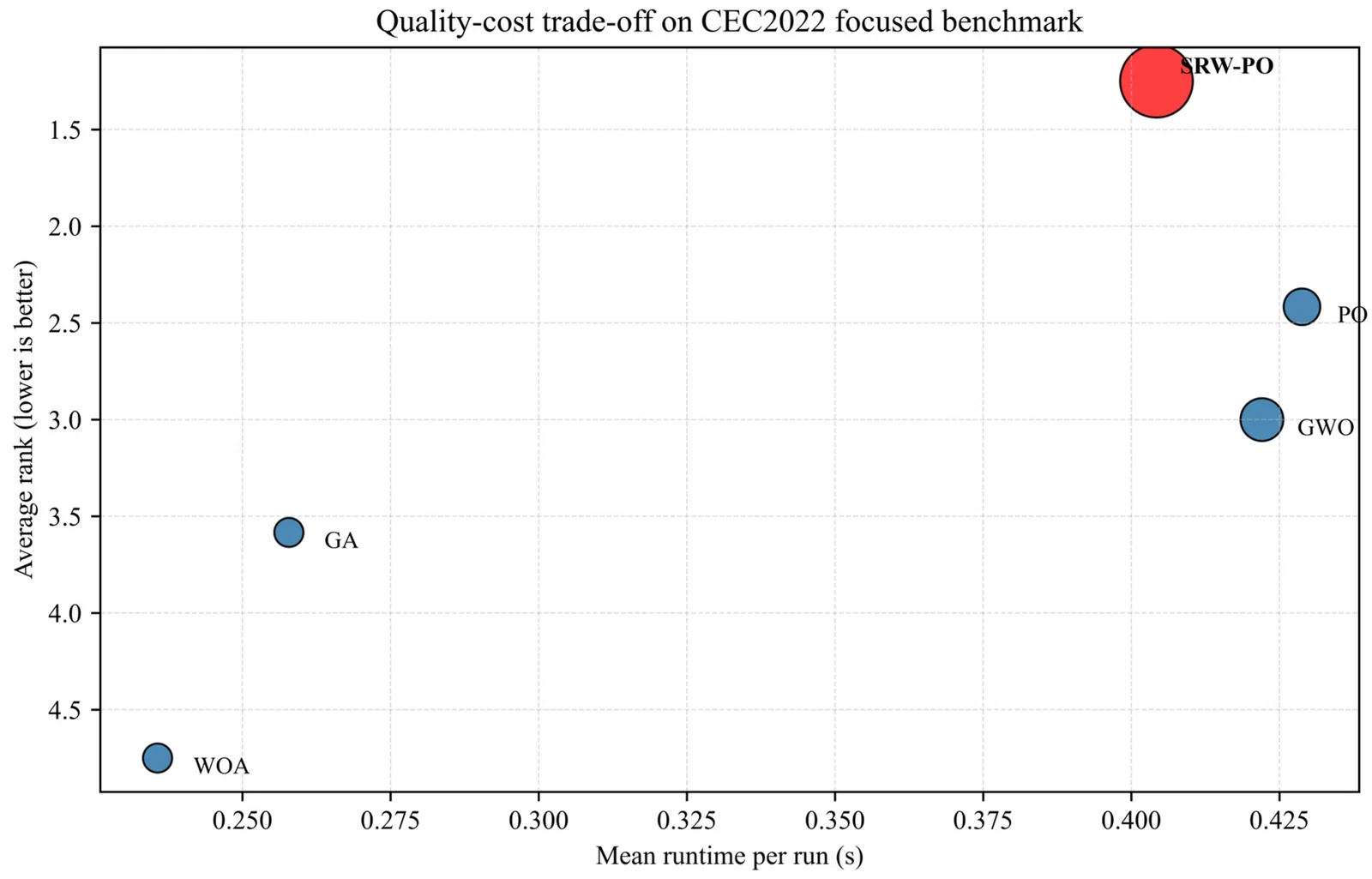
Quality-cost trade-off of the evaluated optimizers on the focused CEC2022 benchmark. The horizontal axis shows the mean runtime per run, and the vertical axis shows the average rank, where lower values indicate better performance. Marker size represents the overall ranking strength of each optimizer across the benchmark functions.

Figure 13 illustrates the quality-cost trade-off of the compared optimizers on the focused CEC2022 benchmark. The horizontal axis represents the mean runtime per run, while the vertical axis shows the average rank, where lower values indicate better performance. Marker size represents the overall ranking strength of each optimizer across the benchmark functions. The proposed SRW-PO appears in the most favorable performance region of the plot, combining the best average rank with a moderate runtime level. The larger marker assigned to SRW-PO indicates that it achieves top-ranking results more frequently across the benchmark functions. Although WOA and GA require shorter runtimes, their average ranks are higher. In contrast, SRW-PO provides a better rank profile than classical PO and GWO at a comparable computational scale. This indicates that the added spiral and random walk operators improve the search outcome without moving the algorithm into a substantially higher runtime regime.

This result supports the use of SRW-PO as the main optimizer for the antenna synthesis experiments. Since array antenna design involves nonlinear objective landscapes with coupled side lobe, null depth, and excitation range constraints, a lower average benchmark rank suggests a stronger general search capability. However, the CEC2022 result should be interpreted as complementary evidence rather than direct proof of radiation pattern improvement. The antenna-specific results in the previous sections provide the direct validation, while Figure 13 shows that the proposed optimizer also maintains a good quality-cost balance on standard numerical benchmark functions.

**Figure 14:**
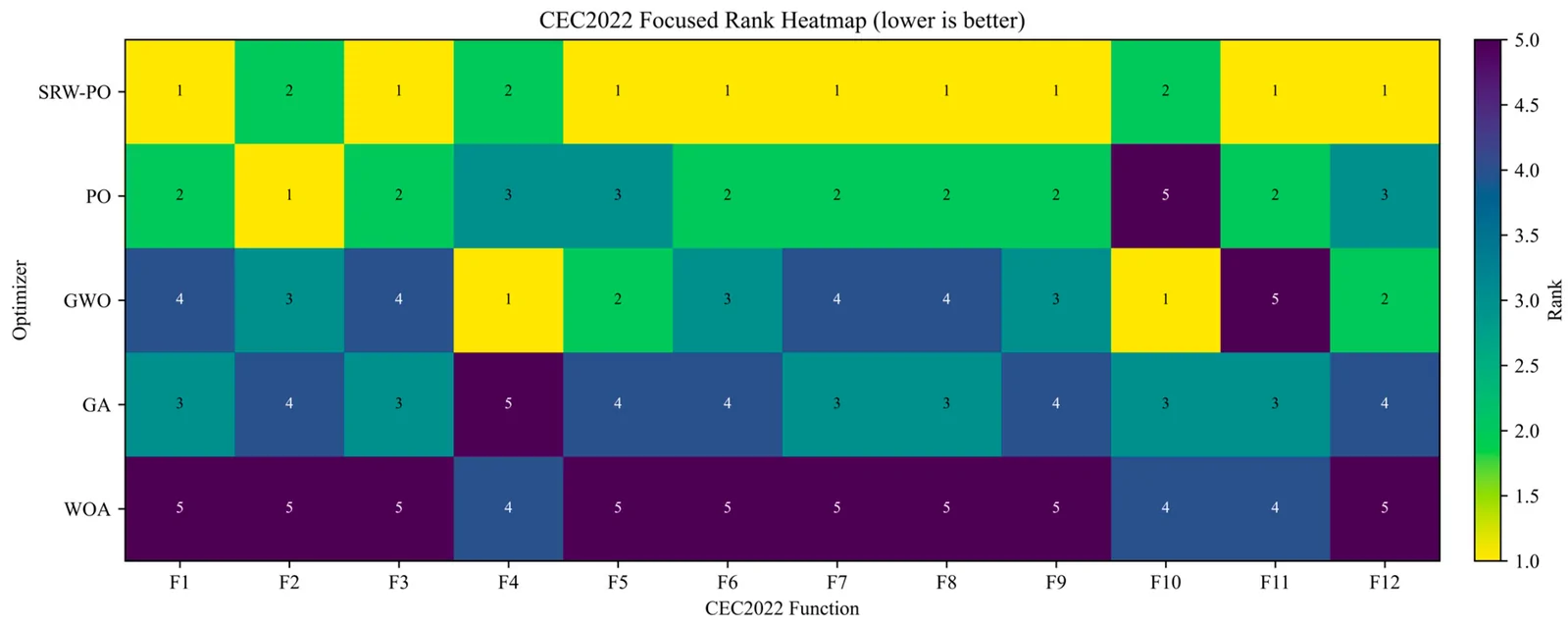
Rank heatmap of the focused CEC2022 benchmark results.

**Figure 15:**
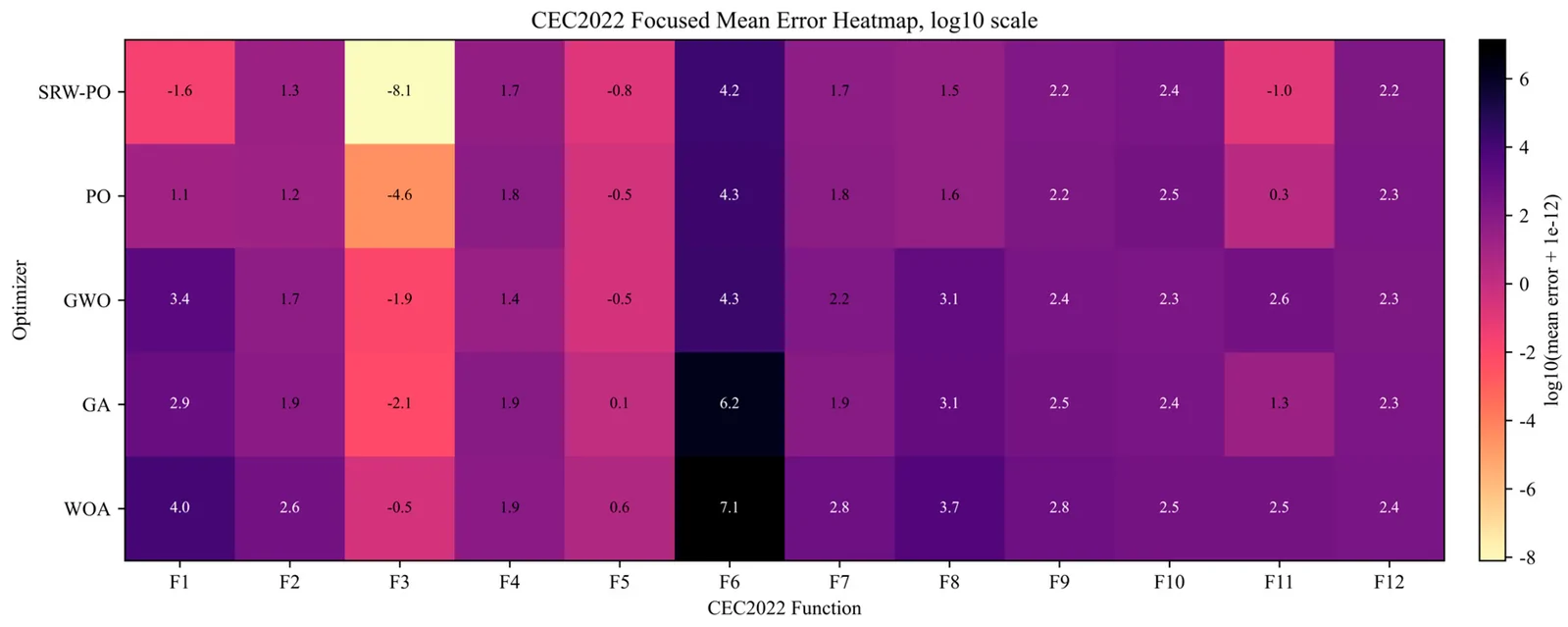
Logarithmic mean error heatmap of the focused CEC2022 benchmark results.

Figure 14 and Figure 15 provide a detailed heatmap-based view of the focused CEC2022 benchmark results. As shown in Figure 14, the proposed SRW-PO achieves the best rank in 9 out of 12 benchmark functions, which demonstrates that its superiority is not limited to a small subset of test cases but is consistently observed across most of the benchmark suite. For the remaining functions, SRW-PO still maintains competitive and promising ranks, indicating that the embedded spiral and random walk mechanisms improve the search process without sacrificing general search consistency. Figure 15 further supports this observation through the logarithmic mean-error distribution. The error profile of SRW-PO remains highly favorable across the benchmark functions, showing that the improved ranking behavior is directly associated with accurate and consistent convergence. These results indicate that SRW-PO provides a balanced behavior between exploration, local refinement, and convergence stability.

### 5.4. Overall Interpretation of Optimization and Radiation Performance

The obtained results show that the proposed SRW-PO optimizer provides an effective radiation pattern synthesis capability under amplitude-only control. The main advantage of the method is its ability to impose deep nulls at prescribed interference directions while preserving the broadside main beam width. In all optimized cases, the HPBW remains fixed at 4.75 deg, indicating that the main beam width is maintained during the optimization process. The optimized patterns demonstrate clear null control capability. In the single-null cases, the achieved null depths range from −95.579 dB to −127.719 dB. In the multiple-null scenarios, all prescribed interference directions are suppressed below approximately −88 dB. This result indicates that SRW-PO can handle both single and multiple interference constraints while keeping the main beam centered at broadside.

The excitation distributions also show an important trade-off between null depth, side lobe level, and amplitude variation. Deep null formation requires redistribution of the excitation amplitudes across the array aperture. However, the obtained DRR values remain bounded in the tested cases. In particular, the two-null and three-null cases achieve DRR values of 5.8320 and 3.3799, respectively, while maintaining deep attenuation at the prescribed angular directions. This indicates that the optimizer does not only search for isolated numerical nulls but also produces excitation distributions that are compatible with amplitude-controlled array synthesis.

With these results, SRW-PO provides a balanced optimization behavior in terms of null placement, main beam preservation, and excitation dynamic range. The results also show that stricter or broader suppression requirements may increase the side lobe level in some cases, which reflects the expected trade-off in amplitude-only array synthesis. Therefore, the proposed method can be considered a suitable optimizer for constrained antenna array synthesis problems where interference suppression, main beam preservation, and excitation range control are treated together.

## 6. Conclusions and Future Work

In this study, an amplitude-only antenna array synthesis approach based on the SRW-PO is presented for side lobe regulation and prescribed null control. The main objective is to obtain deep spatial attenuation at selected interference directions while preserving the broadside main beam behavior and maintaining a feasible excitation distribution.

The proposed method improves the search mechanism of the classical PO by integrating spiral local refinement and adaptive random walk perturbation. The spiral mechanism supports local exploitation around promising solutions, whereas the adaptive random walk strategy increases population diversity and reduces premature convergence tendency. This combined structure provides a balanced optimization process for the nonlinear and constrained nature of antenna array synthesis.

The numerical results show that SRW-PO can generate deep single, multiple, and broad-nulls under amplitude-only control. Across the optimized cases, the HPBW remains fixed at 4.75 deg, indicating that the main beam width is preserved. The achieved null depths range from −79.703 dB to −127.719 dB, while the multiple-null cases suppress all prescribed interference directions below approximately −88 dB. The obtained DRR values also indicate that the optimized excitation amplitudes remain within an acceptable range for amplitude-controlled synthesis.

The main contribution of this work is the development of a metaheuristic synthesis framework that combines deep null placement, main beam preservation, and excitation dynamic range control. Therefore, SRW-PO can be considered a useful optimization alternative for interference-aware linear antenna array design.

Future studies will focus on extending the proposed method to more realistic antenna models, including mutual coupling, embedded element patterns, amplitude quantization, and manufacturing tolerances. In addition, planar array configurations, adaptive beamforming scenarios, and hardware-oriented validation will be investigated to further evaluate the practical applicability of SRW-PO. Scanned beam configurations will also be examined by introducing progressive phase distributions for different steering directions, with particular attention to achievable null depth, side lobe degradation, and scan loss at large steering angles.

## Figures and Tables

**Table 1:** Normalized excitation amplitudes for the synthesized array patterns.

n	Figure 3	Figure 4	Figure 5	Figure 6	Figure 7	Figure 8	Figure 9	Figure 10
0	1.0000	1.0000	1.0000	1.0000	1.0000	1.0000	1.0000	1.0000
+/−1	1.9847	2.8893	2.6546	2.6412	1.8286	1.5857	1.4733	2.6868
+/−2	1.9393	3.8114	3.4387	3.5083	1.8086	1.4194	1.1927	3.3344
+/−3	1.8655	3.4318	3.7758	3.5072	1.8969	1.2256	1.3011	3.1900
+/−4	1.7658	3.1051	2.2803	2.5537	1.7850	1.3880	1.3178	2.6531
+/−5	1.6436	2.6766	3.4059	3.2719	1.4334	1.1693	1.1822	3.3629
+/−6	1.5032	3.1529	2.8285	2.8300	1.4676	1.1446	1.2025	2.5446
+/−7	1.3491	2.8983	2.9152	2.9152	1.4247	1.0395	1.0460	2.9152
+/−8	1.1864	2.6270	2.6440	2.6440	1.4551	0.7427	0.8957	2.5669
+/−9	1.0201	2.2578	2.3668	2.3668	0.8343	0.7468	0.7607	2.3668
+/−10	0.8550	0.7540	0.7583	0.7583	0.6667	0.6451	0.6399	0.7762
+/−11	0.6956	0.4901	0.4928	0.4941	0.3165	0.2719	0.4359	0.5022
+/−12	1.3289	1.5417	1.5482	1.5482	0.9154	0.7530	0.7111	1.5482

**Table 2:** Radiation pattern performance metrics of the representative array synthesis cases.

Figure	MLW	HPBW	NDL	MSL	DRR	Relative Gain Change
Figure 3	12.0	4.75	—	−24.9999	2.8530	—
Figure 4	11.5	4.75	−95.579 @15.50	−17.1655	7.7764	−0.291
Figure 5	11.5	4.75	−99.580 @15.50	−16.1141	7.6626	−0.303
Figure 6	11.5	4.75	−96.760 @15.50	−16.0781	7.1001	−0.266
Figure 7	12.5	4.75	−127.719 @15.50	−21.8273	5.9925	−0.220
Figure 8	13.5	4.75	−94.288 @15.50; −90.394 @25.25	−21.9091	5.8320	−0.265
Figure 9	12.0	4.75	−100.000 @15.50; −89.294 @25.25; −88.136 @36.00	−22.5625	3.3799	−0.126
Figure 10	11.5	4.75	−79.703 @15.50	−16.0791	6.6963	−0.232

**Table 3:** Runtime, function evaluation throughput, and objective cost statistics over 100 independent runs.

Algorithm	Runs	Best Cost	Mean Cost	Std Cost	Mean Time (s)	Mean FE	Mean FE/s
SRW-PO	100	6.8915	**8.0121**	1.0771	**2.2546**	44,449.7100	20,154.1147
PO	100	6.8113	9.4067	11.1254	1.4918	30,030.0000	20,141.3768
GA	100	7.5842	79.0621	76.1489	1.4758	30,030.0000	20,353.3448
WOA	100	7.9976	683.2884	197.3123	1.1980	30,030.0000	25,074.4204
GWO	100	264.2414	468.4545	84.7007	2.8231	30,030.0000	14,946.1149

**Table 4:** Comparative radiation pattern quality metrics achieved by SRW-PO, PO, GA, WOA, and GWO.

Algorithm	Mean MSLL (dB)	Mean Min NDL (dB)	Mean Max NDL (dB)	Mean HPBW	Mean DRR	Best MSLL (dB)	Best Min NDL (dB)	Best Max NDL (dB)
**SRW-PO**	−17.3583	−80.9131	−65.7231	4.7500	7.4797	−16.1282	−80.3915	−65.0004
**PO**	−17.2268	−81.1065	−65.5194	4.7550	7.5417	−18.0823	−80.9873	−67.7767
**GA**	−17.7444	−86.6325	−61.8960	4.7700	7.4980	−16.1270	−78.8965	−65.1616
**WOA**	−17.9729	−66.8394	−39.6592	4.8500	7.0107	−15.4841	−79.5615	−65.0577
**GWO**	−20.1383	−68.5398	−44.4542	4.8500	7.0728	−17.2525	−69.0311	−42.9351

## Data Availability

The data supporting the findings of this study are available from the corresponding author upon reasonable request.

## Abbreviations

The following abbreviations are used in this manuscript:

ABC	Artificial bee colony
CEC2022	2022 IEEE Congress on Evolutionary Computation benchmark suite
DRR	Dynamic range ratio
FE	Function evaluation
FE/s	Function evaluations per second
GA	Genetic algorithm
GWO	Grey wolf optimizer
HHO	Harris hawks optimization
HPBW	Half-power beamwidth
MFO	Moth-flame optimization
MLW	Main lobe width
MSL	Maximum side lobe level
NDL	Null depth level
PO	Puma optimizer
PSO	Particle swarm optimization
SRW-PO	Spiral random walk enhanced puma optimizer
Std	Standard deviation
WOA	Whale optimization algorithm
